# Effect of pH, Temperature, Molecular Weight and Salt Concentration on the Structure and Hydration of Short Poly(*N*,*N*-dimethylaminoethyl methacrylate) Chains in Dilute Aqueous Solutions: A Combined Experimental and Molecular Dynamics Study

**DOI:** 10.3390/polym17162189

**Published:** 2025-08-10

**Authors:** Dimitris G. Mintis, Marco Dompé, Panagiotis D. Kolokathis, Jasper van der Gucht, Antreas Afantitis, Vlasis G. Mavrantzas

**Affiliations:** 1 NovaMechanics Ltd., Nicosia 1070, Cyprus; mintis@novamechanics.com (D.G.M.); afantitis@novamechanics.com (A.A.); 2 Entelos Institute, Nicosia 2102, Cyprus; 3 Physical Chemistry and Soft Matter, Wageningen University, Stippeneng 4, 6708 WE Wageningen, The Netherlands; marcodompe91@gmail.com (M.D.); jasper.vandergucht@wur.nl (J.v.d.G.); 4 NovaMechanics MIKE, 18545 Piraeus, Greece; 5 Department of Chemical Engineering, University of Patras & FORTH-ICE/HT, GR 26504 Patras, Greece; 6 Particle Technology Laboratory, Department of Mechanical and Process Engineering, ETH Zürich, CH-8092 Zürich, Switzerland

**Keywords:** poly(*N*,*N*-dimethylaminoethyl methacrylate), weak polyelectrolyte, LCST behavior, coil–globule structural transition, pH, temperature, salt concentration, molecular weight, radius of gyration, persistence length, pair distribution function, hydrogen bonds

## Abstract

We study the microstructural properties and state of hydration of aqueous low-molecular-weight poly(*N*,*N*-dimethylaminoethyl methacrylate) (PDMAEMA) solutions and their dependence on polymer concentration and pH by means of detailed atomistic Molecular Dynamics (MD) simulations and experiments. For infinitely dilute solutions with a degree of polymerization of *N* = 30 at basic pH conditions, no temperature dependence is observed on the overall shape and state of hydration of the polyelectrolyte. This is supported by the experimental component of our work according to which the hydrodynamic radius, *R*_h_, does not change dramatically with temperature. Small, but not drastic, changes are observed for solutions with longer PDMAEMA chains (*N* = 50, 70, and 110). Although the MD simulations demonstrate that temperature and salt do affect the strength of hydrophobic interactions between PDMAEMA and water, apparently these effects are not strong enough to cause drastic changes to the overall shape of the polymer. MD simulations also reveal that Na^+^ salt ions strongly interact with the oxygen atoms located at the side chain of the polyelectrolyte. While no significant changes in the global shape or state of hydration of the PDMAEMA chain are found, a strong dependence is revealed for the aggregation behavior of the polymer on temperature and salt in slightly more concentrated solutions. A structural transition from a collapsed coil to a stretched conformation is also observed as we move from basic to acidic pH conditions, which is strongly correlated with the degree of chain rigidity as a function of pH.

## 1. Introduction

Polymers undergoing a conformational transition from a coil state to a globule state based on external stimuli are known as “responsive” or “smart” polymers. Their versatile nature favors their use in a wide range of applications including water treatment, tissue engineering, drug delivery, electrophoresis, and separation [1,2]. Poly(*N*,*N*-dimethylaminoethyl methacrylate) (PDMAEMA) is a characteristic example of such a “responsive” or “smart” polymer which shows large reversible conformational changes with temperature and pH. Water-soluble thermo-responsive polymers exhibiting a low critical solution temperature (LCST) behavior find great applicability in biological applications [3,4], a field where PDMAEMA attracts remarkable attention due to its high antibacterial and fungicidal activity enabling its usage in the development of antimicrobial coatings [5,6,7,8,9]. In addition, PDMAEMA is reported to be among the most effective polymers considered to carry genetic material (e.g., DNA or RNA) into cells with high transfection efficiency and low toxicity [10,11,12].

Polymers exhibiting LCST behavior are expected to undergo a coil-to-globule conformational transition as the solution temperature increases above the LCST [13]. Hydrogen bonds between the polymer chain and water molecules are weakened until they break, resulting in an increase in hydrophobic interactions and, in turn, causing aggregation and phase separation of the polymer [14]. Despite being a thermo-responsive polymer, PDMAEMA is a weak polyelectrolyte whose behavior may critically be dictated by pH changes. At acidic pH, the PDMAEMA chain is heavily positively charged, whereas at basic pH it remains uncharged. As a result, PDMAEMA solutions exhibit much higher LCSTs at acidic pH (due to high electrostatic intrachain repulsions) than at basic pH conditions [15,16]. Addressing the synergetic effect of hydrophobic and electrostatic interactions on the behavior of PDMAEMA solutions is one of the main goals of the present study.

The LCST behavior of PDMAEMA solutions and their dependence on pH, salt concentration, and polymer molecular weight *M*_w_ has been investigated by several groups [15,16,17], and rather consistent predictions have been reported. However, very little is known so far about the microstructural features of relatively short PDMAEMA chains, which are the focus of the present study. In the context of this work, we will restrict our literature review to studies that have considered dilute PDMAEMA solutions (a total polymer concentration of ~ 1 wt%) at basic pH conditions where electrostatic interactions are not of major concern. Bütün and co-workers [17] reported that the LCSTs of PDMAEMA solutions with *M*_w_s between 1.45 and 53 kDa vary from 46.4 to 32.2 °C. Macias and co-workers [15] found that for PDMAEMA solutions with *M*_w_s in the range between 2.6 and 45.0 kDa, the LCSTs vary from around 54 to 25 °C, while Mohammadi and co-workers [16] found that the LCSTs of PDMAEMA solutions with *M*_w_s in the range between 5.5 and 85.8 kDa vary from around 74 to 25 °C. Small discrepancies are therefore noted regarding the exact values of the LCSTs of PDMAEMA solutions, and this may be attributed to the accuracy and efficiency of the analytical technique used in the experimental measurements which typically include turbidimetry [18], also known as the cloud-point technique; differential scanning calorimetry (DSC) [19]; dynamic light scattering (DLS) [20]; nuclear magnetic resonance (NMR) [21]; and infrared (IR) spectroscopy [22].

As far as simulations are concerned, so far and to the best of our knowledge, only two all-atom Molecular Dynamics (MD) studies [23,24] have been reported on the coil-to-globule transition of PDMAEMA. They have been carried out with a single 30-monomer-long alternately protonated (corresponding to neutral pH conditions) PDMAEMA chain at infinite dilution across a wide range of temperatures (from 280 to 360 K). Although the term infinite dilution is commonly used in the literature to describe single-chain simulations in dilute solutions, the term ultra dilute may offer a more precise description as the infinite dilution term technically refers to the limit where the polymer’s concentration approaches zero. However, for consistency with previous studies [25,26], we retain this terminology also in the present work. In both studies, a coil-to-globule transition was observed at 338 K as was evident from the reduction in the radius of gyration of the chain from a value approximately equal to 16 Å (at 283 and 303 K) to a value equal to 11 Å at 338 K. However, both studies employed relatively short simulation times, between 30 and 50 ns, which may limit the reliability of the conclusions drawn. In a very recent all-atom MD study [27], much longer simulation times were accessed, on the order of 200 ns, for alternately protonated 30 mer PDMAEMA chain brushes to examine how their properties are influenced by variations in grafting density, temperature, and pH. Individual alternately protonated 30 mer PDMAEMA threads (not confined in space) were also studied, and a very minor decrease (only by 1 Å at 338 K) was observed for the radius of gyration compared to the value (approximately 16 Å) at 300 K. The discrepancies noticed between different studies highlight the need for truly long simulations that can reliably capture the thermo-responsive structural transitions of PDMAEMA; at the same time, they also underline the importance of conducting laboratory experiments to support and validate the simulation findings. Our aim here is to address both gaps by performing very long all-atom MD simulations, exceeding 250 ns, for PDMAEMA solutions with molecular lengths ranging from 30 to 110 monomers, both in infinitely dilute and semidilute solutions, at three different ionization states (0%, 50%, and 100%) across a range of temperatures and ionic strengths (salt concentrations), together with conducting experiments to support the simulation results.

Since only very few MD studies have been conducted so far on the folding behavior of a single PDMAEMA chain in dilute solutions, we also review below the most noteworthy simulation works with other types of thermo-responsive polymers (of similar chain length to the ones considered here) in dilute solutions. One such very well-known polymer is poly(*N*-isopropylacrylamide) (PNIPAM), and many atomistic MD simulations have already been carried out to study systems of single PNIPAM chains in aqueous solutions [28,29,30]. Alaghemandi and Spohr [28] reported no change in the global shape and state of hydration of PNIPAM with *N* < 30 as the temperature was increased above the LCST. This observation was also observed in another MD study [31] in which no change in the conformation and hydration of a PNIPAM chain with *N* = 3, 5, or 10 was observed. On the contrary, Alaghemandi and Spohr [28] observed that the mean value of the radius of gyration of the PNIPAM chain with *N* = 30 decreased by approximately 8 Å as the temperature increased above the LCST. A similar observation was reported by de Oliveira and co-workers who studied a single PNIPAM chain with *N* = 32 [29]. Du and co-workers [30] observed that the conformational state and hydration behavior of PNIPAM with *N* = 50 were greatly affected by the increase in temperature above the LCST. Overall, these studies suggest that systems consisting of relatively short single thermo-responsive chains in aqueous solutions do not exhibit a profound coil-to-globule transition. This statement is also supported by a different MD study [32] where the behavior of a single poly(2-[2-methoxyethoxy]ethyl methacrylate) (PMEO2MA) chain with *N* = 5, 20, and 50 in an aqueous solution was examined, and no conformational changes were recorded for *N* = 5 and *N* = 20, while for *N* = 50 the mean value of the radius of gyration decreased by approximately 5 Å as the temperature increased above the LCST [32]. Of course, since the thermo-responsive polymers discussed here do not exhibit a pH response, the analogy with PDMAEMA should be taken with caution.

As far as ionic strength effects are concerned, several studies [33,34,35] have reported that ion-specific effects can play a prominent role in the LCST behavior of thermo-responsive macromolecular solutions. Idziak and co-workers [36] found that the addition of sodium chloride to a poly (*N*,*N*-diethylacrylamide) (PDEA) solution caused a linear decrease in its LCST. The effect of different types of salt ions (Hofmeister effects) [37] and salt concentration on the LCST behavior of PNIPAM has also been investigated both by experiments [33,38] and MD simulations [30,39]. Earlier reports [33,38] suggested that salt anions play a critical role in the LCST conformational transition of PNIPAM, since it was believed that they form direct bonds with the amide groups on the polymer side chains. However, two MD works performed by Du and co-workers [30] and Heyda and co-workers [39] elucidated that salt cations have stronger affinity with the oxygen atom located at the side chain of the polymer, while salt anions have no association with the polymer. Salt anions have been reported to affect polymer stability only via the salt cation–anion interaction [30]. It is worth pointing out that Zhang and co-workers [38] in their experimental studies observed that PNIPAM exhibits a single LCST that is independent of molecular weight and salt concentration in the presence of weakly hydrated (chaotropic) anions such as Cl^−^ and Br^−^.

There has been no other computational or experimental study so far that has fully addressed the microstructural features of short PDMAEMA chains, ranging from 30 to 110 monomer units, as a function of temperature and salt concentration in dilute aqueous solutions. The objective of the present work is to carry out detailed atomistic MD simulations and validate predictions with experiments to shed light on the effect of temperature on the microstructure of PDMAEMA (with chain length *N* = 30–110) at basic pH conditions in quite dilute solutions. It is important, however, to clarify that we do not intend to determine here the LCST of PDMAEMA solutions, as this would require a more detailed and systematic thermodynamic approach. On the other hand, in our study here we also wish to shed light on the effect of salt concentration (only NaCl is considered as salt) on the structural stability of the PDMAEMA chain. Moreover, we carry out MD simulations to study hydration, chain local stiffness and rigidity, and chain global conformation as a function of pH (at 0, 50, and 100% ionization states) for systems consisting of a single PDMAEMA chain with a molecular length of *N* = 30. The accuracy of the molecular mechanics force field employed in our study has already been examined and documented in a previous study [40] where predictions for the conformational and dynamical behavior of PDMAEMA in aqueous solutions were found to be in excellent agreement with measured data.

This work is structured as follows: Section 2 presents a detailed description of the simulated systems and of the experiments performed. Section 3 presents our results and discussions regarding the effect of temperature and ionic strength on the microstructure and state of hydration of PDMAEMA solutions for various chain lengths at basic pH conditions. The effects of polymer–salt interactions and pH on the local rigidity, global shape, and hydration of PDMAEMA are also discussed in this section. We conclude with Section 4 outlining the most important findings of our work and highlighting possible future research plans and directions.

## 2. Materials and Methods

### 2.1. Experiments

Two samples of atactic PDMAEMA with respective number average molecular weights *M*_n_s equal to 5.5 and 19.0 kg mol^−1^ (degrees of polymerization *N* = 33 and 120, respectively) were purchased from Polymer Source, Inc. (Montreal, QC, Canada). The corresponding polydispersity indices were 1.08 and 1.34. With both samples, PDMAEMA aqueous solutions with concentrations spanning from 9 to 33 kg m^−3^ were prepared by dissolving PDMAEMA in Milli-Q water. The exact concentrations of the solutions are reported in Table 1.

The relative viscosities of the aqueous solutions were measured at temperatures *T* = 20 and 50 °C using a Ubbelohde viscometer immersed in a water bath. A heating plate was placed below the heating bath, and the temperature was controlled with a thermocouple. After introducing the aqueous solution into the instrument’s reservoir, the liquid was sucked through a capillary and a measuring bulb. When the pressure was released, the liquid traveled back through the bulb. The relative viscosity was obtained by measuring the time it takes the liquid to pass through two calibrated marks using the following equation:

(1) $$\eta_{\mathrm{rel}}=\frac{\eta }{\eta_{0}}=\frac{t}{t_{0}}$$

where *η*_rel_ denotes the relative viscosity (Pa s), *η* is the viscosity of the polymer solution (Pa s), *η*_0_ is the viscosity of the solvent (Pa s), *t* is the time it takes the polymer solution to pass through the two marks (s), and *t*_0_ is the time it takes the solvent to pass through the two marks (s). Every measurement was performed in triplicate. The aqueous solution containing PDMAEMA with the highest molecular weight (*M*_n_ = 19.0 kg mol^−1^) turned white when it was heated to 50 °C, indicating macroscopic phase separation; in this case, the aforementioned equation is not valid, and no data were collected.

### 2.2. MD Simulations

A list of all simulated systems studied in this work together with all relevant technical details (such as molecular mechanics force field employed, the number of polymer chains *n*_chains_ considered, the charge fitting method, chain length *N*, temperature *T* (K), salt concentration *c*_salt_ (M), the water model, the degree of ionization *α*^+^ (%), and the total simulation time (ns)) is presented in Table 2. Simulation times varied across systems depending on the chain length, system size, and time required to reach equilibrium. Longer simulations were performed only when it was deemed necessary to ensure complete equilibration.

Atactic stereochemistry was adopted in all cases, and the generalized AMBER force field (GAFF) was chosen to describe intra- and intermolecular interactions. Numerical values for the parameters controlling bonded and non-bonded contributions to the total potential energy of the system at the 50 and 100% protonation states were borrowed from our previous MD study [40]. For the case of 0% ionization, parametrized atomic partial charges were obtained based on the restrained electrostatic potential (RESP) [41] method, using as input the electronic densities obtained from single-point energy calculations at the Hartree–Fock level of theory with the 6-31G* basis set. The GAMESS-US (version 2016R1) software [42,43] was used to perform the quantum mechanical calculations for the electronic densities. Section S1 of the Supporting Information Section of this manuscript provides a detailed description of the assigned atomic partial charges for the case of 0% ionization. The corresponding numerical values of the parameters regarding the bonded contributions and the Lennard–Jones parameters *σ* and *ε* at 0% ionization were also borrowed from our previous MD study [40]. Note that PDMAEMA at basic pH is unprotonated (0% ionized); at neutral pH, it is partially protonated (50% ionized), and at acidic pH, it is fully protonated (100% ionized). For water, the SPC/E molecular model was considered. The Lennard–Jones parameters for Na^+^ and Cl^−^ ions needed to model ion–ion, ion–water, and ion–polymer interactions in the solution were obtained from the work by Joung and Cheatham [44]. Additionally, and for the purpose of force field validation, the OPLS [45], MMFF [46], and PCFF [47] force fields for PDMAEMA at the 50% ionization state were implemented and utilized, along with the charge method and water model, as listed in Table 2.

**Figure 1:**
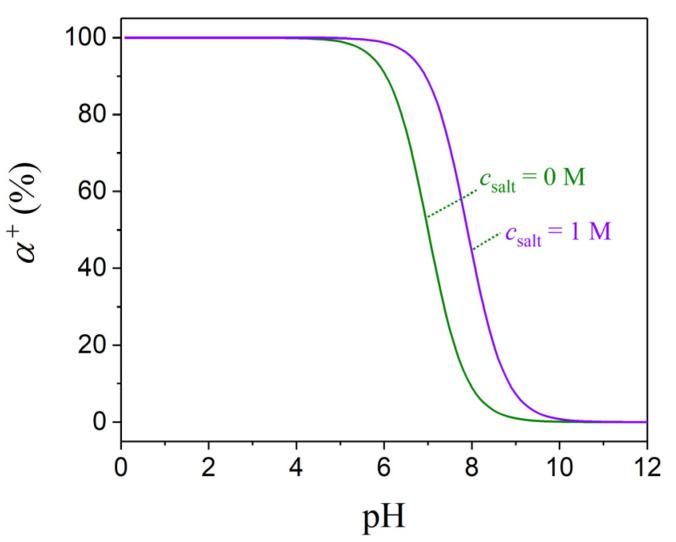
Degree of ionization α^+^ of PDMAEMA as a function of pH at two different salt concentrations (0 and 1 M).

Accurately representing the degree of ionization for PDMAEMA is very critical, as this is influenced not only by pH but also by other factors such as ionic strength (salt concentration), salt type, polymer concentration, solvent quality, and temperature [48]. In the case of PDMAEMA, a weak polybase, the protonation behavior can be well approximated with the Henderson–Hasselbalch equation:

(2) $$\alpha^{+}=\frac{10^{pK_{b}-\mathrm{pH}}}{1+10^{pK_{b}-\mathrm{pH}}}$$

where the base dissociation constant p*K*_b_ determines the shape and position of the titration curve. Experimental studies have reported a p*K*_b_ value of 7.3 ± 0.1 under zero salt conditions [49], while at a 1 M salt concentration, this value shifts to approximately 7.9, as can be confirmed through empirical fitting to titration data [48,50]. As shown in Figure 1, by plotting the degree of ionization as a function of pH at 0 and 1 M salt concentrations, it is found that at pH = 3, PDMAEMA is fully protonated under both conditions; at pH = 7, the degree of ionization is around 50% at a 0 M salt concentration and approximately 90% at a 1 M salt concentration; and at pH values above 10, the polymer is essentially uncharged. While constant-pH MD simulations are capable of capturing such pH-dependent transitions by dynamically adjusting the protonation state of the titratable groups during the simulation [51,52,53,54], these methods are computationally intensive and require either Monte Carlo sampling [55] or λ-dynamics schemes [56]. To reduce the computational cost, the solvent is often treated as a dielectric continuum that assumes equilibrium with the solute at each protonation step. In this study, we chose to adopt conventional MD simulations, where the protonation state is kept fixed during the run. This approximation is valid for the fully protonated and fully deprotonated cases; for the partially protonated one, we adopted an alternating protonation scheme along the polymer backbone, a common practice in earlier simulations of weak polyelectrolytes under neutral pH conditions [24]. Notably, in our simulations at a 1 M salt concentration, we retained this alternating protonation pattern to represent the 50% ionization level. While this corresponds to a pH of approximately 7.9 (rather than to strictly neutral pH), this simplification was made to ensure systematic comparability across simulation conditions. The approach captures representative ionization scenarios and is consistent with literature-supported protonation behavior of PDMAEMA across acidic, neutral, and basic pH environments [24,25].

All MD simulations of this work were performed with GROMACS (version 2016.3) [57], except for the system where the PCFF force field was used for both PDMAEMA and water molecules which was simulated with LAMMPS (version 31Mar17) [58]. Initial configurations were constructed with the MAPS software by Scienomics (MAPS platform, version 4.2, Scienomics SARL, Paris, France) using a modified configurational bias Monte Carlo scheme [59,60]. The same simulation protocol was followed for all systems studied in this work. For example, each system was initially subjected to a potential energy minimization scheme to get rid of any overlaps between atoms. This was followed by a short simulation in the nVT statistical ensemble (*n* denotes the total number of interacting units in the simulation cell, *V* the volume of the cell, and *T* the temperature); then production MD runs were conducted in the nPT statistical ensemble (*P* denotes the pressure) for the total simulation time, as presented in Table 2. A cubic simulation cell subject to periodic boundary conditions (pbc’s) in all three directions was employed for all systems. Also, sufficiently large simulation cells were constructed (approximately two times larger than the end-to-end distance of the PDMAEMA chain) to ensure the complete absence of any spurious interactions between atomistic units and their periodic images in neighboring cells. For the minimization of potential energy, the steepest-descent algorithm was employed, with the criterion for energy convergence set to 50 kJ mol^−1^ nm^−1^. The leap-frog algorithm for the integration of the microscopic equations of motion was employed for the simulations in the nVT statistical ensemble for a total simulation time of 1 ns with an integration time step of 1 fs. The temperature was maintained at its constant value with the help of a Nosé–Hoover thermostat [61,62], with the coupling constant being equal to 1 ps. The nPT production runs were conducted using the leap-frog integrator with an integration time step of 2 fs and by constraining all bonds with LINCS [63]. In addition to the Nosé–Hoover thermostat, a Parrinello–Rahman barostat [64] was also to maintain a constant pressure according to the desired value (*P* = 1 atm). The Particle Mesh Ewald (PME) [65] method was used to deal with long-range electrostatic interactions.

## 3. Results and Discussion

A rigorous analysis of the molecular mechanics force field employed in our work to describe intra- and intermolecular interactions of a PDMAEMA chain (at a 100% degree of ionization) has already been carried out in our previous MD study [40], in which it was revealed that the GAFF force field in combination with atomic partial charges computed with the RESP charge fitting method can capture the conformational and dynamical behavior of PDMAEMA solutions with very good accuracy compared to experimentally measured data. In the next paragraphs, we present additional validation of the force field and of the model constructed in the present work for the case of an alternately protonated PDMAEMA chain by comparing against previous all-atom MD studies [23,24].

A detailed description of the method used to calculate average values from the MD simulations has already been provided in our previous works [25,26,40,66]. In particular, for the calculation of the statistical error for all equilibrium averages, the standard deviation of the instantaneous values from the average value was used except for the statistical error for the persistence length, *L*_p_, which was computed using block averaging. In our experimental work, on the other hand, we monitored the phase transition of the PDMAEMA solutions and calculated the relative viscosities of the solutions by means of a capillary viscometer.

### 3.1. Force Field Validation

As already discussed in the Introduction Section, two MD studies have so far investigated the coil-to-globule transition of a single, alternately protonated PDMAEMA chain in infinitely dilute solutions, but they relied on relatively short simulation times (between 30 and 50 ns). In this section, we reproduced the Min et al. [24] system by employing the PCFF force field for both PDMAEMA and water molecules. As already explained above, this MD simulation was conducted using LAMMPS [58]. We also simulated the same system with the GAFF, OPLS, and MMFF force fields along with the RESP [41] method for assigning atomic partial charges at 338 K where the transition from the coil-to-globule state is reported, using GROMACS [57]. Again, as already mentioned earlier, water molecules were simulated with the SPC/E model.

**Figure 2:**
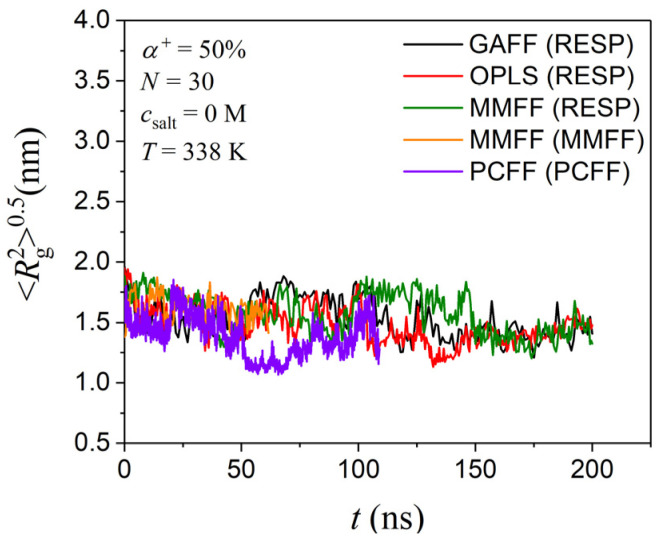
Time evolution of the radius of gyration of an alternately protonated 30 mer long PDMAEMA chain at 338 K and a 0 M salt concentration from the present simulations with all different force fields (GAFF, OPLS, MMFF, and PCFF).

The time evolution of the radius of gyration is reported in Figure 2. The system with the PCFF force field demonstrates a decrease in the value of the radius of gyration from 16 Å to around 11 Å at 338 K, consistent with the findings reported by Min et al. [24] and Nagumo et al. [23]. As the simulations are extended to longer times, the radius of gyration does not seem to remain constant; instead, it fluctuates. For a single alternately protonated 30 mer PDMAEMA chain at 338 K at infinite dilution, the equilibrium value of the radius of gyration appears to be between 15 and 16 Å. This finding is consistent with the findings of the very recent study by Tippner et al. [27], where the radius of gyration of individual alternately protonated 30 mer PDMAEMA threads (not confined in space) is approximately 15 Å. For their study, Tippner et al. [27] conducted 200 ns long all-atom MD simulations using the GAFF2 force field for PDMAEMA along with the RESP method for assigning atomic partial charges. The latter had been derived from density functional theory (DFT) calculations performed at the B3LYP/def2-SVP level. Therefore, the coil-to-globule transition for an alternately protonated 30 mer PDMAEMA chain at infinite dilution at 338 K reported previously [23,24] cannot be confirmed, which also emphasizes the importance of conducting sufficiently long simulations to extract firm conclusions. For the subsequent analysis in this work, the GAFF force field along with the RESP method was used for PDMAEMA, and the SPC/E model was used for water molecules.

### 3.2. The Effects of Chain Length, Temperature, and Salt Concentration on an Unprotonated PDMAEMA Chain

The state of hydration and the conformational properties of a single PDMAEMA chain at infinite dilution under basic pH conditions (equivalent to an unprotonated PDMAEMA chain) in the temperature range between 277 and 370 K at two different salt concentrations (0 and 1 M) and for different chain lengths (*N* = 30, 50, 70, and 110) are presented in this section.

#### 3.2.1. Chain Length

**Figure 3:**
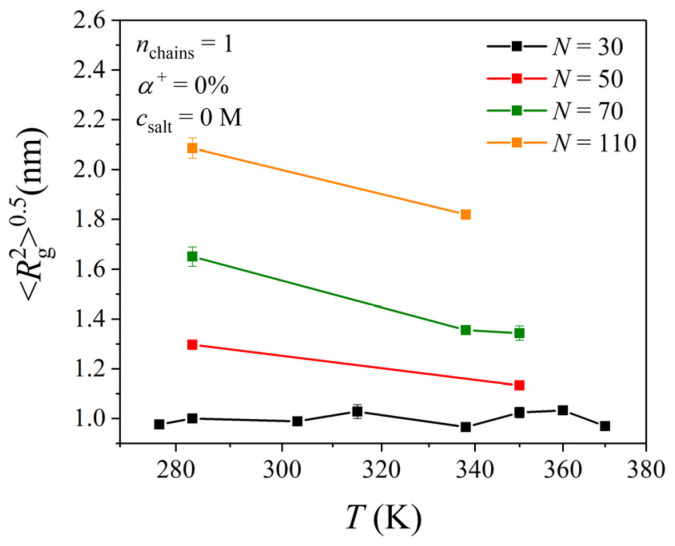
MD predictions for the average radius of gyration of a PDMAEMA chain as a function of temperature for the four different chain lengths considered here (corresponding to degrees of polymerization equal to *N* = 30, 50, 70, and 110).

Figure 3 presents the MD predictions for the average radius of gyration, ${\langle R_{g}^{2}\rangle }^{0.5}$, of a single unprotonated PDMAEMA chain with varying chain length in a salt-free solution. The time evolution of ${\langle R_{g}^{2}\rangle }^{0.5}$ is reported in Section S2 (Supporting Information). It is observed that the value of ${\langle R_{g}^{2}\rangle }^{0.5}$ of the shortest PDMAEMA chain (*N* = 30) does not vary with temperature, which suggests that its global shape does not experience any structural transition but instead remains at a globule state. Similar findings had been reported by Min et al. [24]. On the other hand, for the PDMAEMA chain with *N* = 50, a slight (albeit not drastic) difference in the value of ${\langle R_{g}^{2}\rangle }^{0.5}$ (by approximately 2 Å) is observed as the temperature increases from 283 to 338 K. A similar decrease in the value of ${\langle R_{g}^{2}\rangle }^{0.5}$ (by approximately 3 Å) is observed for the PDMAEMA chain with *N* = 70 as the temperature increases from 283 to 338 K. For the PDMAEMA chain with *N* = 110, this difference is around 3.6 Å.

In previous MD studies [28,30,31] of single PNIPAM chains with *N* < 30 in aqueous solutions, no conformational dependency on temperature had been observed. A similar behavior had been reported in another MD study with a single poly(2-[2-methoxyethoxy]ethyl methacrylate) (PMEO_2_MA) chain: no conformational changes were recorded for chains with *N* = 5 and 20 [32]. These observations are in line with our predictions here for the single unprotonated PDMAEMA chain with *N* = 30. However, for longer PNIPAM chains, the ${\langle R_{g}^{2}\rangle }^{0.5}$ value was found to decrease by approximately 8–10 Å with temperature [29,30,31]. In other MD studies [67,68] the ${\langle R_{g}^{2}\rangle }^{0.5}$ of a single poly(*N*-vinylcaprolactame) (PVCL) chain with *N* = 30 and *N* = 100 in an aqueous solution was found to decrease by approximately 8 Å with temperature. Our present study suggests much milder changes in ${\langle R_{g}^{2}\rangle }^{0.5}$ with temperature for single unprotonated PDMAEMA chains with *N* > 30 (by approximately 1–4 Å).

**Figure 4:**
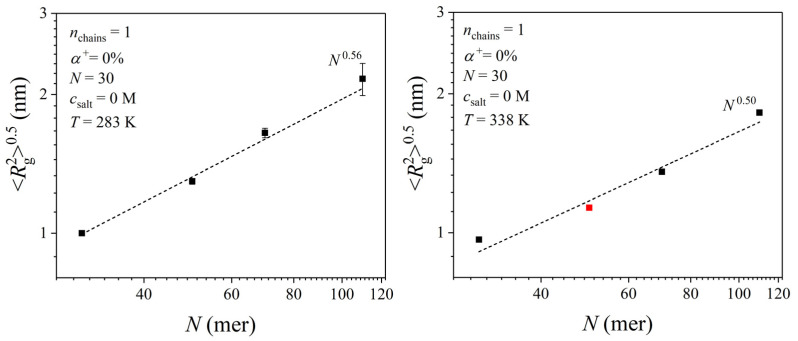
Scaling of the radius of gyration with degree of polymerization for a 0%-ionized PDMAEMA chain at two different temperatures: 283 and 338 K. The red data point for *N* = 50 is from a simulation at 350 K and has been included in the comparison for consistency (a slight deviation is expected due to the higher temperature).

To further understand the conformational behavior of PDMAEMA chains with a 0% degree of ionization, we examined the scaling relationship between the radius of gyration and the degree of polymerization at 283 and 338 K. This is shown in Figure 4, revealing a power-law scaling of the form ${\langle R_{g}^{2}\rangle }^{0.5}∼N^{v}$, with the Flory exponent being equal to *ν* = 0.56 at 283 K and equal to *ν* = 0.50 at 338 K. The data point for *N* = 50 (indicated with a red dot) corresponds to 350 K and is included for consistency in the comparison, and a minor deviation may be expected due to the higher temperature. Nevertheless, the above values indicate that PDMAEMA adopts a nearly ideal random coil conformation below and above the LCST. This finding suggests that uncharged PDMAEMA remains in a swollen state at both temperatures, unlike other weak polyelectrolytes such as poly(acrylic acid) (PAA) and poly(ethylene imine) (PEI), where more collapsed conformations have been observed under similar uncharged conditions [25,26]. This difference may be attributed to the unique chemical structure of PDMAEMA, which contains a longer and more flexible side chain with an ester group and a dimethylamino group. These structural characteristics may enhance its hydrophobicity, which can result in a more expanded chain conformation compared to PAA and PEI.

Next, we computed the Kuhn length *b* and the corresponding number of Kuhn segments *N*_K_ per chain for all PDMAEMA chains investigated in this work (30, 50, 70, and 110 mer) using the relationships *L* = *N*_K_·*b* and *b* = *R*_ee_^2^/*L*, where *L* is the contour length and *R*_ee_ is the end-to-end distance [69]. The results are summarized below in Table 3. The 30 mer chain, in particular, is characterized by ~14 Kuhn segments. In general, polymer chains with *N*_K_ < 30 lack sufficient inherent flexibility [70,71]. Thus, the 30 mer chain, due to its high inherent rigidity, may not have enough conformational freedom to undergo a sharp coil-to-globule transition. In contrast, the 110 mer chain is characterized by *N*_K_~40, indicating increased flexibility.

**Figure 5:**
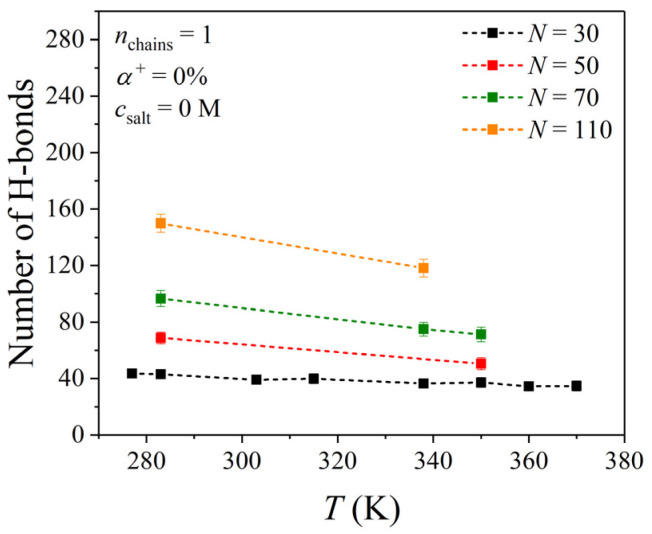
MD predictions for the total number of hydrogen bonds formed between the PDMAEMA chain and water molecules as a function of temperature. Results for different chain lengths equivalent to *N* = 30, 50, 70, and 110.

The state of hydration of a PDMAEMA chain was also studied by quantifying the total number of hydrogen bonds formed between the chain and water molecules, and it was examined as a function of temperature (for chain lengths corresponding to *N* = 30, 50, 70, and 110). As can be seen from Figure 5 and similar to the behavior of ${\langle R_{g}^{2}\rangle }^{0.5}$, no obvious variation is observed for the chain with the shortest chain length (*N* = 30). On the hand, for the PDMAEMA chains with *N* = 50, 70, and 110, a slight decrease is observed, which is again consistent with the behavior of ${\langle R_{g}^{2}\rangle }^{0.5}$. The time evolution of the number of hydrogen bonds formed between the PDMAEMA chain and water molecules for all systems considered in our study is reported in Section S3 of the Supporting Information Section. In addition to this, the surface area of the PDMAEMA chain which is accessible to water molecules (SASA) is quantified and traced as a function of time (see Section S4 of the Supporting Information Section).

**Figure 6:**
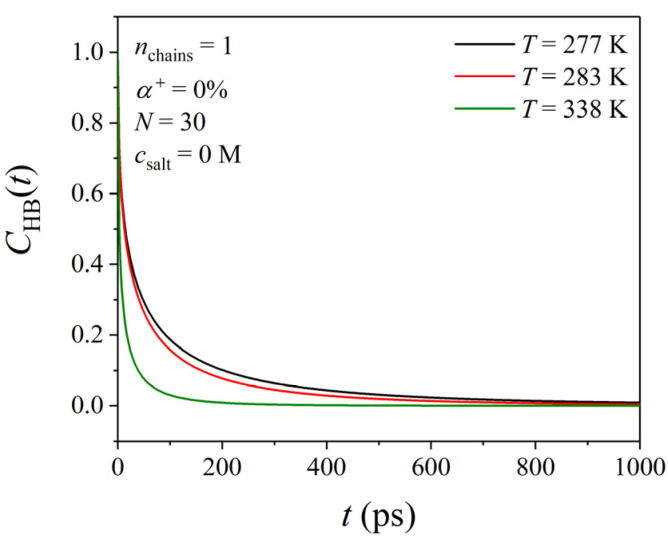
Decay of the function $C_{\mathrm{HB}}\left(t\right)$ with time *t* for the PDMAEMA chain with *N* = 30 in the absence of salt at three different temperatures (*T* = 277, 283, and 338 K).

We also explored the effect of temperature on the strength (lifetime) of hydrogen bonds. The lifetime of hydrogen bonds is expressed as the time *τ*_BF_ for the bound-to-free transition computed from the time decay of the autocorrelation function $C_{\mathrm{HB}}\left(t\right)=\frac{\langle h_{ij}\left(t\right)h_{ij}\left(0\right)\rangle }{\langle h_{ij}{\left(0\right)}^{2}\rangle }≅\exp\left(-\frac{t}{\tau_{\mathrm{BF}}}\right)$ (in the long time limit) where *h_ij_* (*t*) takes the value of one if molecules *i* and *j* are bonded with a hydrogen bond at the time interval (0, *t*) and the value of zero otherwise. The activation energy *E*_A_ for such a transition is also computed from the corresponding expression of transition-state theory, namely $\frac{1}{\tau_{\mathrm{BF}}}=\frac{k_{B}T}{h}e^{-E_{A}/k_{B}T}$, where *k*_B_ is the Boltzmann constant and *h* the Planck constant. According to Figure 6, the *C*_HB_ decays to zero considerably faster for the PDMAEMA solution at *T* = 338 K than for the solutions at *T* = 277 and 283 K. In addition, for the lifetimes of hydrogen bonds (see Table 3), we see that $\tau_{\mathrm{BF}}\left(T=283\;\;K\right)>\tau_{\mathrm{BF}}\left(T=338\;\;K\right)$, suggesting that as the temperature increases, the strength of hydrogen bonds weakens (implying an increase in the strength of hydrophobic interactions between PDMAEMA and water molecules).

#### 3.2.2. Salt Concentration

As far as the ionic strength is concerned, our MD simulations reveal that $\tau_{\mathrm{BF}}\left(c_{\mathrm{salt}}=0\;\;M\right)>\tau_{\mathrm{BF}}\left(c_{\mathrm{salt}}=1\;\;M\right)$ and $E_{A}\left(c_{\mathrm{salt}}=0\;\;M\right)>E_{A}\left(c_{\mathrm{salt}}=1\;\;M\right)$ (see Table 4), which indicates that salt does affect the strength of hydrogen bonds.

**Figure 7:**
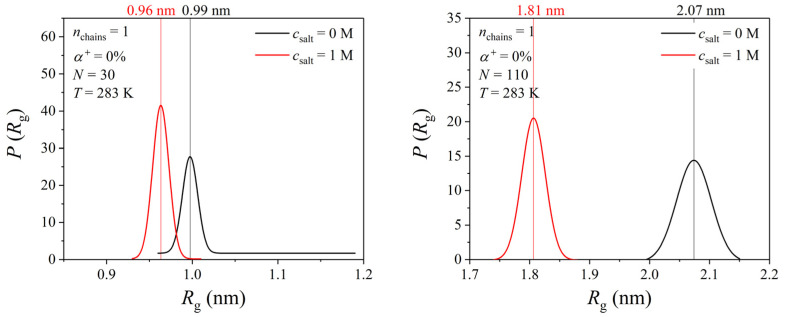
MD predictions for the probability density distribution of the radius of gyration of a single PDMAEMA chain with *N* = 30 and 110 in an aqueous solution at two different salt concentrations (0 and 1 M).

To further elaborate on the effect of ionic strength, we computed the probability density distribution of the radius of gyration at 0 and 1 M salt (NaCl) concentrations for the systems consisting of a single PDMAEMA chain with lengths of *N* = 30 and 110. Figure 7 suggests only a negligible effect of salt on the global shape of the chain with *N* = 30 but a slight decrease in the radius of gyration (by about 2.6 Å) for the chain with *N* = 110.

From our MD simulation results so far, we conclude that for systems consisting of a single unprotonated PDMAEMA chain with a rather short chain length (*N* = 30), no appreciable conformational changes take place with temperature. However, some effects are observed on the strength of the hydrogen bonds formed between the polymer and water molecules as a function of temperature and salt. This effect is stronger for the unprotonated PDMAEMA chain with *N* = 50, 70, and 110, but the differences are not as drastic as one would have expected for a thermo-responsive polymer exhibiting LCST behavior. To elucidate this point further, we performed viscosity experiments, which can help us study the phase behavior (and thus examine possible structural transitions) of PDMAEMA solutions. The measurements were performed with two PDMAEMA samples with *N* = 33 and 120 at two temperatures (293 and 333 K) for a total of five polymer concentrations in the range between 1 and 3 wt% (dilute concentration regime). No phase transition was revealed for the PDMAEMA solution with *N* = 33 as the temperature increased from 293 to 333 K (the solution remained transparent). On the contrary, for the solution with *N* = 120, a macroscopic phase transition was observed as the temperature increased from 293 to 333 K (the solution turned turbid).

From the viscosity measurements, we quantified the hydrodynamic radius of the polymer by employing the Mark–Houwink relation for dilute polymer solutions:

(3) $$\left(\eta_{\mathrm{rel}}-1\right)/c_{p}=2.5N_{A}\left(\frac{4}{3}\pi R_{h}^{3}/M_{n}\right)$$

where *N*_A_ denotes the Avogadro constant and *R*_h_ denotes the hydrodynamic radius. Computed *R*_h_ values for the PDMAEMA solution with *N* = 33 did not vary significantly with temperature (only a small decrease by about 1 Å was observed as the temperature increased from 293 to 333 K). Also, as already commented in Section 2.1, the *R*_h_ of a PDMAEMA solution with *N* = 120 at 333 K could not be measured because the polymer underwent phase separation; thus the above approach for calculating *R*_h_ was not valid.

**Figure 8:**
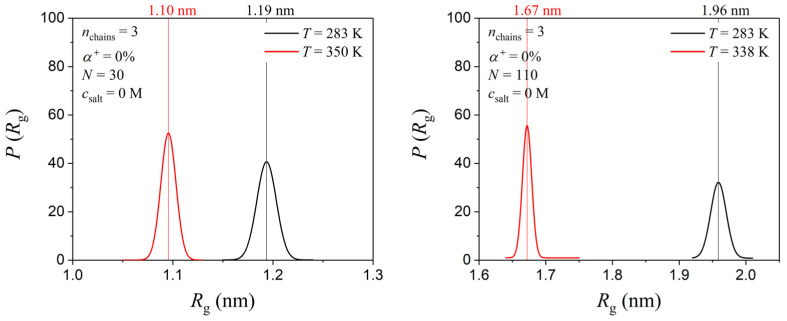
MD-predicted probability density distributions of the PDMAEMA chain radius of gyration at two different temperatures for the PDMAEMA solutions at 2 and 3 wt% total polymer concentrations, with *N* = 30 and 110, respectively.

To ensure absolute consistency with the experimental measurements, further MD simulations were carried out by considering two additional systems: one containing PDMAEMA with *N* = 30 at a 2 wt% total polymer concentration (equivalent to a total of three PDMAEMA chains in the simulation volume) and a second one containing PDMAEMA with *N* = 110 at 3 wt% (equivalent to a total of three PDMAEMA chains in the simulation volume). As observed in Figure 8, for the solution with *N* = 30, the value of the radius of gyration decreases by about 0.9 Å as the temperature increases from 283 to 350 K. This is in full agreement with our experimental measurements which indicated a decrease in *R*_h_ by about 1 Å. Regarding the PDMAEMA chain with *N* = 110, the radius of gyration decreases by about 3 Å as the temperature increases from 283 to 338 K, which is similar to what we had already observed for the single-chain system. Note, however, that the Mark–Houwink relationship is ideally applicable under dilute conditions while our experimental concentration range here extends up to 3 wt%, which approaches the theoretical overlap concentration for PDMAEMA chains (around 3 wt%). While intrinsic viscosities were obtained by extrapolating to a concentration of zero to mitigate interchain effects, slight deviations from ideal dilute behavior may still be present at the highest concentrations studied.

#### 3.2.3. PDMAEMA Aggregation and Its Dependence on Temperature and Salt

**Figure 9:**
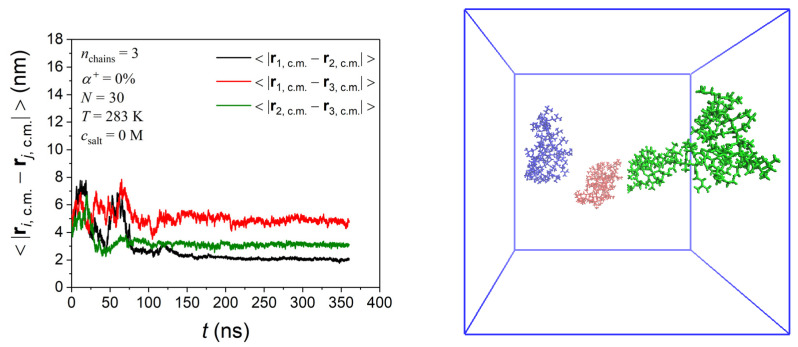

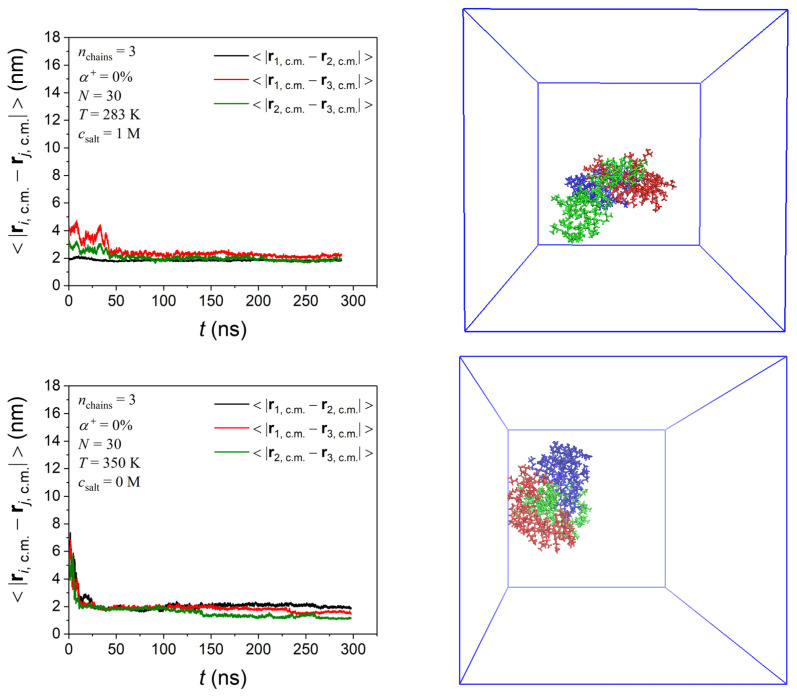
MD predictions for the temperature and ionic strength dependence of the aggregation behavior in the PDMAEMA solutions with *N* = 30. The left panel provides a measure of the distance between the centers of mass of a pair of PDMAEMA chains. The right panel shows a characteristic snapshot of the final, equilibrated configuration.

Next, we examine how PDMAEMA chain aggregation depends on temperature and salt for the multi-chain solutions. Figure 9 presents the results for two systems containing three PDMAEMA chains with *N* = 30 at *T* = 283 and 350 K in salt-free solutions, as well as for a third one containing three PDMAEMA chains with *N* = 30 at 283 K in a solution with a 1 M salt concentration. Characteristic snapshots of the final equilibrated configurations as well as the measure of the distance between the centers of mass of two PDMAEMA chains are also included. For the PDMAEMA solution at 283 K with no salt, the PDMAEMA chains do not feel any mutual attraction; thus, they remain at distances larger than 2 nm apart one from the other. On the other hand, at the same temperature but in the 1 M salt solution, PDMAEMA chains tend to form an aggregate throughout the simulation, which provides direct evidence that polymer–polymer interactions depend strongly on ionic strength. Aggregation of PDMAEMA chains is also observed for the PDMAEMA solution at 350 K with no salt. We can therefore argue that PDMAEMA solutions with *N* = 30 exhibit both strong temperature- and ionic strength-dependent aggregation characteristics. The strong temperature-dependent aggregation behavior is also observed for the PDMAEMA solutions with *N* = 110 (see Section S5 of the Supporting Information Section).

To further elaborate on the effect of temperature on the rigidity or flexibility of PDAMEMA, we computed the quasi-harmonic entropy (*S*_QH_) for a quantum–mechanical harmonic oscillator as being a measure of the configurational (or conformational) entropy of a PDMAEMA chain [72]:

(4) $$S_{\mathrm{QH}}=k_{B}\sum_{i}^{3N-6}\left(\frac{\hbar \omega_{i}/k_{B}T}{e^{\hbar \omega_{i}/k_{B}T}-1}-\ln\left(1-e^{-\hbar \omega_{i}/k_{B}T}\right)\right)$$

In Equation (4), ℏ=h/2π while $\omega_{i}={\left(k_{B}T/\lambda_{i}\right)}^{1/2}$ is the angular frequency corresponding to the eigenvalue *λ_i_* obtained by diagonalizing the mass-weighted variance–covariance matrix, ***C***, of PDMAEMA atomic positions as recorded in the course of the MD simulations. The sum in Equation (4) runs over all 3N−6 nonzero *λ_i_* eigenvalues of this mass-weighted variance–covariance matrix ***C***. The 3N×3N elements of matrix ***C*** after roto-translational superposition of individual simulated structures are given as

(5) $$C_{ij}=\langle M_{i}^{1/2}\left(r_{i}-\langle r_{i}\rangle \right)M_{j}^{1/2}\left(r_{j}-\langle r_{j}\rangle \right)\rangle$$

where 1≤i≤3N and 1≤j≤3N. In Equation (5), ***M*** is the atomic mass matrix and ***r*** denotes the position vector of a PDMAEMA atom, and the average is taken over all structures stored in the trajectory file. Our implementation of the quasi-harmonic entropy equation has been validated against a previous computational study [73] for the configurational entropy of an unbound ubiquitin structure (PDB code: 1UBQ) [74] (see Figure S7 of the Supporting Information Section).

**Figure 10:**
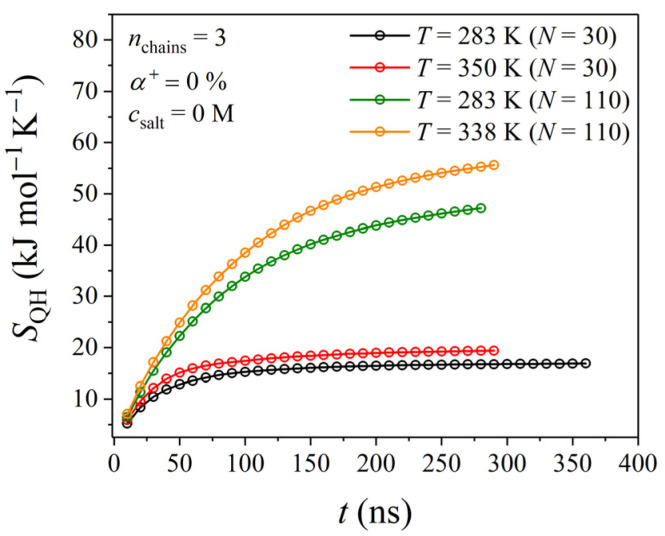
Time evolution of the configurational entropy of a PDMAEMA chain as a function of temperature and chain length.

By definition, the configurational entropy is proportional to the logarithm of the number of different conformational states that give the same observable result (for example, the same end-to-end distance). Therefore, flexible polymer chains would have higher configurational entropy than rigid polymers. As can be seen from Figure 10, and despite the fact that the calculations of the configurational entropies for the systems consisting of PDMAEMA chains with *N* = 110 have not fully converged even after 250 ns, the configurational entropy of PDMAEMA above the LCST is slightly higher than the corresponding one below the LCST. This denotes that PDMAEMA chains are characterized by slightly higher flexibility above the LCST, which can be explained by the fact that structural constraints become weaker as the temperature increases; in contrast, below the LCST, PDMAEMA chains adopt rather rigid conformations characterized by a lower degree of flexibility.

Based on our MD predictions so far, no variation is observed in the microstructural features and state of hydration of a single, short (*N* = 30) unprotonated PDMAEMA chain in an aqueous solution. This was confirmed by the experimental component of our work which revealed that the hydrodynamic radius, *R*_h_, of the PDMAEMA chain with *N* = 30 remains almost unchanged upon changing the temperature. On the contrary, for a single unprotonated PDMAEMA chain with *N* = 50, 70, and 110, small changes occur, which become more pronounced as the number of chains in the system increases. The overall picture therefore suggests that the conformational characteristics of an unprotonated single PDMAEMA chain at basic pH conditions remain unchanged with temperature (due to its highly hydrophobic nature), and the only dependence with temperature and salt arises when the concentration of the solution increases.

#### 3.2.4. Salt Ions and Their Interaction with PDMAEMA

**Figure 11:**
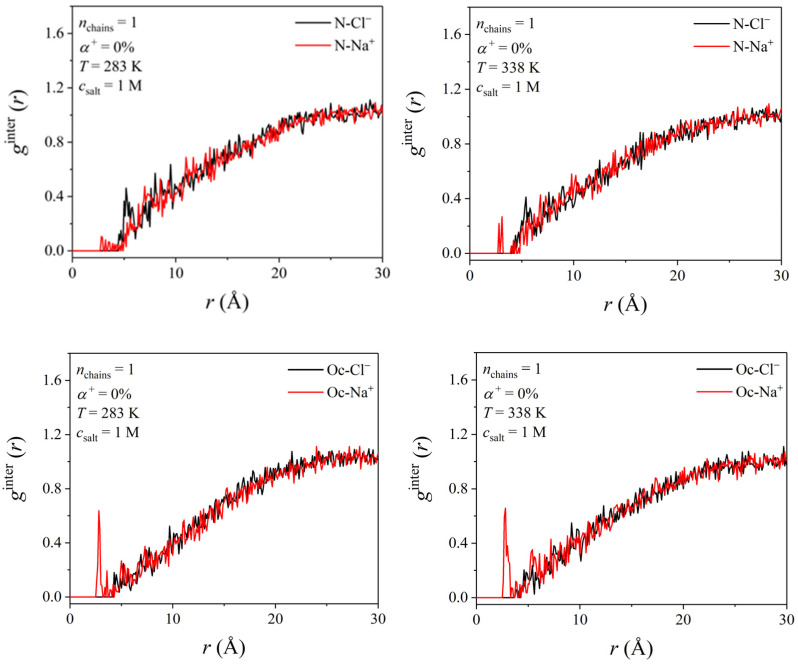
Radial intermolecular pair distribution function of salt anions (Cl^−^) and salt cations (Na^+^) with the reference atoms N and Oc on the side chain of a PDMAEMA chain at two different temperatures (*T* = 283 and 338 K).

As already discussed in the Introduction Section, previous studies [33,34,35,36,37,38] have reported the critical role of salt ions in the LCST behavior of a thermo-responsive polymer. Thus, it is essential to microscopically address the interaction between salt ions (Na^+^ and Cl^−^ here) and the PDMAEMA chain. Figure 11 shows the radial intermolecular pair distribution function, *g*^inter^ (*r*), of Cl^−^ and Na^+^ ions with the reference atoms N and Oc on the side chain of an unprotonated PDMAEMA chain (please check Figure S8 of the Supporting Information Section for the notation of these atoms) in a 1 M salt concentration solution at 283 and 338 K. The relevant radial intermolecular pair distribution function, *g*^inter^ (*r*), of Cl^−^ and Na^+^ with the reference atoms Cb and Cn of the PDMAEMA chain is provided in Section S7.1 of the Supporting Information Section. All *g*^inter^ (*r*) curves reported in this study have been obtained by averaging over the last 20–30 ns of the simulation using a binning width equal to 0.1 Å.

According to Figure 11 and for all temperatures studied, Na^+^ cations are closer to the N (distance approximately equal to 2.8 Å) and Oc (distance approximately equal to 2.6 Å) atoms of the PDMAEMA chain in comparison to Cl^−^ anions. In addition, the *g*^inter^ (*r*) curves for Oc-Na^+^ pairs exhibit a higher peak than for N-Na^+^ pairs, indicating a stronger interaction between Na^+^ ions and the Oc atoms located on the side chain of the polymer rather than with the N atoms located in the termini of the side chains. Our observation is in excellent agreement with the previous MD studies by Du and co-workers [30] and Heyda and co-workers [39] for PNIPAM, where salt cations were found to have stronger affinity with the oxygen atoms located on the side chain of the polymer while salt anions show no association with the polymer.

### 3.3. The Effect of pH and Salt Concentration

**Figure 12:**
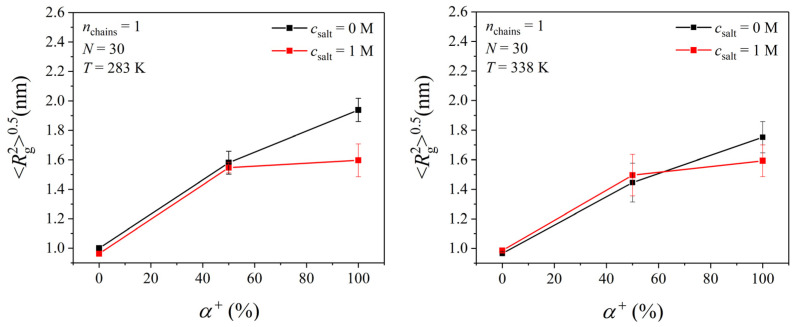
MD-predicted ${\langle R_{g}^{2}\rangle }^{0.5}$ values of a PDMAEMA chain with *N* = 30 at two different temperatures (*T* = 283 and 338 K) and two different salt concentrations (c_salt_ = 0 and 1 M) for the three different degrees of ionization (α^+^ = 0, 50, and 100%) considered in this work.

We conducted further MD simulations to study the effect of pH (equivalently, the degree of ionization of the PDMAEMA chain) and ionic strength on the microstructure and state of hydration of PDMAEMA solutions with *N* = 30, at 283 and 338 K. Only systems consisting of a single PDMAEMA chain (infinite dilution) were considered here. Figure 12 presents the ${\langle R_{g}^{2}\rangle }^{0.5}$ value of such a PDMAEMA chain at the two different temperatures (*T* = 283 and 338 K) and two different salt concentrations (*c*_salt_ = 0 and 1 M) for the three different degrees of ionization considered (*α*^+^ = 0, 50, and 100%). As already discussed above, the ${\langle R_{g}^{2}\rangle }^{0.5}$ value of an unprotonated PDMAEMA chain with *N* = 30 does not vary with temperature and salt concentration. However, as the degree of ionization increases, the ${\langle R_{g}^{2}\rangle }^{0.5}$ value increases drastically, implying a structural transition of the PDMAEMA chain from a highly collapsed conformation to a stretched conformation with an increasing degree of ionization regardless of the temperature and salt concentration. No obvious effect of temperature is observed on the ${\langle R_{g}^{2}\rangle }^{0.5}$ value for the 50- and 100%-ionized PDMAEMA chains. Worth discussing is the result at 283 K, where as the salt concentration increases from 0 to 1 M, the ${\langle R_{g}^{2}\rangle }^{0.5}$ value decreases from around 2 nm to 1.6 nm. This shows that the presence of salt also affects the shape of a fully charged PDMAEMA chain at 283 K.

**Figure 13:**
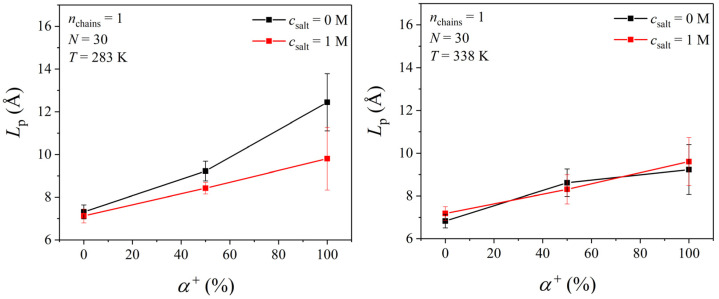
MD-predicted *L*_p_ values of a PDMAEMA chain with *N* = 30 at two different temperatures (*T* = 283 and 338 K) and two different salt concentrations (c_salt_ = 0 and 1 M) for the three different degrees of ionization (α^+^ = 0, 50, and 100%) considered in this work.

In a next step, we computed the persistence length, *L*_p_, of the PDMAEMA chain, and the results obtained are shown in Figure 13, revealing a similar trend to that of the dependence of ${\langle R_{g}^{2}\rangle }^{0.5}$ on the degree of ionization. The persistence length, *L*_p_, is obtained from the mean value of the projection of the end-to-end vector **R**_ee_ on the direction of any bond **I***_i_* along the chain backbone (and averaging over all such bonds):

(6) $$L_{p}=\frac{1}{N_{b}}\sum_{i=1}^{N_{b}}\frac{\langle R_{ee}\cdot l_{i}\rangle }{\left|l_{i}\right|}$$

where *N*_b_ denotes the total number of bonds along the chain backbone. The persistence length of a PDMAEMA chain is found to drastically increase as the degree of ionization increases, indicating that the rigidity of the chain also defines the global shape of the polymer. The presence of salt (1 M) also affects the local rigidity of the chain at 283 K. The increase in persistence length with the degree of ionization is primarily attributed to enhanced intrachain electrostatic repulsions between the protonated tertiary amine groups located on the side chain of the PDMAEMA. These repulsions drive the chain into more extended conformations, effectively reducing local flexibility and increasing chain stiffness, a behavior already discussed in the literature in the context of theoretical models for polyelectrolytes [75,76]. Additionally, hydration effects also contribute significantly. As ionization increases, the polymer becomes more hydrophilic, and the surrounding water molecules form stronger solvation shells around the charged groups, further stabilizing extended conformations and hindering intramolecular folding; this contributes further to the observed increase in rigidity [76]. Our observations here align with previous MD simulations with linear and branched poly(ethylene imine) (PEI) [26] and poly(acrylic acid) [25], where an increase in the degree of ionization of the polyelectrolyte was accompanied by an increase in its persistence length; this underscores the combined effect of electrostatic repulsion and hydration on chain rigidity.

**Figure 14:**
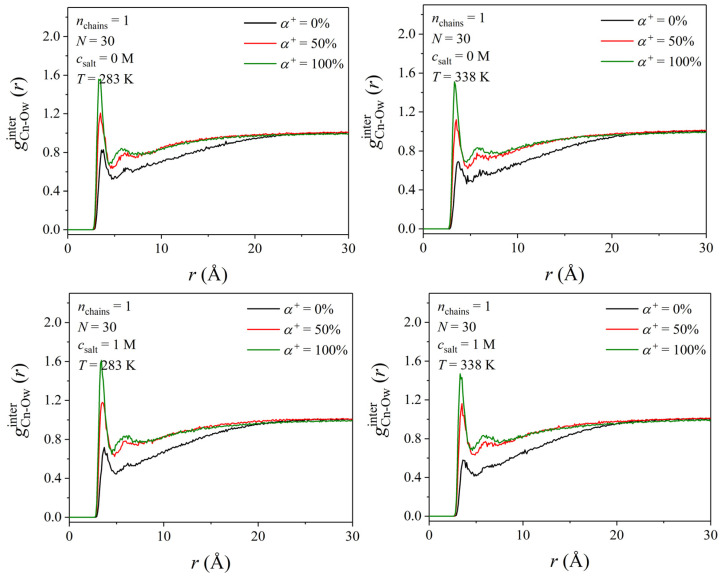
Effect of the degree of ionization on the radial distribution function of Cn-Ow pairs at two different temperatures (*T* = 283 and 338 K) and two different salt concentrations (*c*_salt_ = 0 and 1 M) for the three different degrees of ionization (*α*^+^ = 0, 50, and 100%) considered in this work.

We also computed the intermolecular radial pair distribution function, *g*^inter^ (*r*), between polymer and water molecules, which, among others, can help identify the value of the minimum interaction distance between polymer atoms and water molecules. Figure 14 presents the *g*^inter^ (*r*) between the Cn atom on the PDMAEMA chain and the Ow atom of water molecules. The analysis between the pairs Cb-Ow, Oc-Ow, and N-Ow is given in Section S7.2 of the Supporting Information Section. Clearly, as the degree of ionization increases, the strength of the interaction between water oxygens and the Cn, Cb, Oc, and N atoms of PDMAEMA chain increases considerably, regardless of the temperature and salt concentration. Notably, at 100% ionization, a higher $g^{\mathrm{inter}}\left(r\right)$ intensity value is observed than at 50% ionization, suggesting stronger interactions between the fully protonated PDMAEMA chain and water molecules. The minimum distance of interaction between the Cn atom and the Ow atom does not seem to change considerably with temperature and salt concentration. However, the intensity of the *g*^inter^ (*r*) for the unprotonated PDMAEMA chain decreases with the salt concentration, thus denoting that upon the addition of salt, the strength of interaction becomes weaker for both systems at 283 and 338 K. This observation is in line with our estimates of the lifetime of hydrogen bonds formed between polymer and water molecules (upon the addition of salt, the lifetime of hydrogen bonds becomes shorter). No such obvious salt effect is observed for the PDMAEMA chain at the 50 and 100% ionization states.

## 4. Conclusions

In this work, long fully atomistic MD simulations in combination with experiments were performed to examine the effect of temperature on the microstructure and state of hydration of aqueous dilute PDMAEMA solutions with molecular lengths of *N* = 30, 50, 70, and 110 under basic pH conditions. The effect of ionic strength was also examined. Only NaCl was considered in this study as salt. We also studied the effect of pH on the global shape and hydration of PDMAEMA solutions with a chain length of *N* = 30.

Thermo-responsive polymers exhibiting LCST behavior are generally expected to show a notable structural coil-to-globule transition. The present study suggests no such drastic structural transition for a single, short (*N* = 30) unprotonated PDMAEMA chain with temperature, as well as some small changes for longer chains (*N* = 50, 70, and 110). To clarify this point, viscosity measurements were performed for PDMAEMA solutions with two chain lengths (*N* = 33 and 120) at two temperatures, one above and one below the LCST. The measurements showed that the hydrodynamic radius of the PDMAEMA solution with *N* = 30 does not change drastically as the temperature increases. The inherent rigidity of short PDMAEMA chains, such as the 30 mer chain studied here, likely hinders the coil-to-globule transition, as evidenced by the low number of Kuhn segments (~14) and low configurational entropy (~15 kJ mol^−1^ K^−1^) characterizing such a chain. Notably, a 0%-ionized PDMAEMA chain exhibited near-ideal random coil behavior at both temperatures (above and below its LCST), unlike PAA and PEI [25,26], which have been reported to assume collapsed conformations under similar conditions.

MD simulations further revealed that the strength of hydrogen bonds formed between an unprotonated PDMAEMA chain with *N* = 30 and water molecules weaken with increasing temperature or when salt is added to the solution. Our findings suggest that no changes take place in terms of the local rigidity, global shape, and hydration of short PDMAEMA chains at infinite dilution under basic pH conditions; this is due to the highly hydrophobic nature of an unprotonated PDMAEMA chain. On the other hand, strong aggregation dependence on temperature and salt was observed when the concentration of PDMAEMA in the solution increased. Calculations of the configurational entropy indicated that PDMAEMA chains are characterized by higher flexibility above the LCST. The authors of this article, however, point out the need for a more thorough and systematic investigation of other molecular–mechanics force fields as well as other techniques of assigning atomic partial charges for a more in-depth investigation of the LCST behavior of PDMAEMA solutions in the context of fully atomistic MD simulations.

The value of pH was found to dramatically alter chain rigidity, global shape, and the hydration behavior of PDMAEMA solutions with a chain length of *N* = 30. As the ionization of the PDMAEMA chain was increased, its persistence length, *L*_p_, was found to increase considerably. Also, the global shape of the PDMAEMA chain, quantified through its average radius of gyration, was found to increase with the degree of ionization. A strong correlation between the global shape of the PDMAEMA chain and solvation was obtained. Fully charged PDMAEMA chains were found to be highly soluble, while un-protonated PDMAEMA chains were found to be highly hydrophobic.

In the future we intend to systematically address the effect of chain length and temperature on both alternately protonated and fully protonated, single PDMAEMA chains at infinite dilution, as we conducted here for unprotonated chains. We also plan to examine the effect of salt ion type and concentration on the microstructural properties and state of hydration of PDMAEMA solutions at different pH conditions.

## Figures and Tables

**Table 1:** List of PDMAEMA solutions investigated experimentally and their concentration.

Sample	*c*_1_ (kg m^−3^)	*c*_2_ (kg m^−3^)	*c*_3_ (kg m^−3^)	*c*_4_ (kg m^−3^)	*c*_5_ (kg m^−3^)
PDMAEMA *M*_n_ = 5.5 kg mol^−1^	9.98	14.42	18.53	25.95	32.44
PDMAEMA *M*_n_ = 19.0 kg mol^−1^	9.68	13.99	17.98	25.18	31.47

**Table 2:** List of all simulated systems conducted in this work and some technical details about them.

No	*n*_chains_	*N* (mer)	*c*_salt_ (M)	*T* (K)	Force Field	Charge Method	H_2_O Model	Degree of Ionization, *α*^+^ (%)	Total Simulation Time (ns)
1	1	30	0	277	GAFF	RESP	SPCE	0%	549
2	1	30	0	283	GAFF	RESP	SPCE	0%	822
3	1	30	0	303	GAFF	RESP	SPCE	0%	315
4	1	30	0	315	GAFF	RESP	SPCE	0%	705
5	1	30	0	338	GAFF	RESP	SPCE	0%	490
6	1	30	1	283	GAFF	RESP	SPCE	0%	494
7	1	30	1	338	GAFF	RESP	SPCE	0%	300
8	1	30	0	350	GAFF	RESP	SPCE	0%	239
9	1	30	0	360	GAFF	RESP	SPCE	0%	234
10	1	30	0	370	GAFF	RESP	SPCE	0%	232
11	3	30	0	283	GAFF	RESP	SPCE	0%	360
12	3	30	0	350	GAFF	RESP	SPCE	0%	297
13	3	30	1	283	GAFF	RESP	SPCE	0%	287
14	1	50	0	283	GAFF	RESP	SPCE	0%	322
15	1	50	0	350	GAFF	RESP	SPCE	0%	372
16	1	70	0	283	GAFF	RESP	SPCE	0%	378
17	1	70	0	338	GAFF	RESP	SPCE	0%	327
18	1	70	0	350	GAFF	RESP	SPCE	0%	170
19	1	110	0	283	GAFF	RESP	SPCE	0%	746
20	1	110	0	338	GAFF	RESP	SPCE	0%	286
21	3	110	0	283	GAFF	RESP	SPCE	0%	285
22	3	110	0	338	GAFF	RESP	SPCE	0%	296
23	1	70	1	283	GAFF	RESP	SPCE	0%	383
24	1	70	1	338	GAFF	RESP	SPCE	0%	332
25	1	110	1	283	GAFF	RESP	SPCE	0%	300
26	1	110	1	338	GAFF	RESP	SPCE	0%	304
27	1	30	0	283	GAFF	RESP	SPCE	50%	200
28	1	30	0	303	GAFF	RESP	SPCE	50%	200
29	1	30	0	338	GAFF	RESP	SPCE	50%	200
30	1	30	1	283	GAFF	RESP	SPCE	50%	200
31	1	30	1	303	GAFF	RESP	SPCE	50%	200
32	1	30	1	338	GAFF	RESP	SPCE	50%	200
33	1	30	0	338	OPLS	RESP	SPCE	50%	200
34	1	30	0	338	MMFF	RESP	SPCE	50%	200
35	1	30	0	338	MMFF	MMFF	SPCE	50%	61
36	1	30	0	338	PCFF	PCFF	PCFF	50%	109
37	1	30	0	283	GAFF	RESP	SPCE	100%	300
38	1	30	0	338	GAFF	RESP	SPCE	100%	300
39	1	30	1	283	GAFF	RESP	SPCE	100%	300
40	1	30	1	338	GAFF	RESP	SPCE	100%	300

**Table 3:** Estimated Kuhn segment properties of PDMAEMA chains as a function of molecular length (*N* = 30, 50, 70, and 110).

*N* (mer)	30	50	70	110
*L* (nm)	9.1	15.2	21.3	33.6
*b* (nm)	0.66	0.67	0.77	0.83
*N*_K_	13.7	22.8	27.9	40.4

**Table 4:** Lifetime of hydrogen bonds as a function of temperature and the corresponding activation energy for the PDMAEMA chain with *N* = 30 at infinite dilution in water at 0 and 1 M salt concentrations.

$T\left(K\right)$	$\tau_{\mathrm{BF}}\left(\mathrm{ps}\right)$		$E_{A}\left(\mathrm{kcal}\;\;{\mathrm{mol}}^{-1}\right)$	
	$c_{\mathrm{salt}}=0\;\;M$	$c_{\mathrm{salt}}=1\;\;M$	$c_{\mathrm{salt}}=0\;\;M$	$c_{\mathrm{salt}}=1\;\;M$
277	32.54		3.14	
283	32.51	21.29	3.14	2.89
338	5.10	3.20	2.05	1.77

## Data Availability

Topology files for modeling poly(*N*,*N*-dimethylaminoethyl methacrylate) at different ionization states using GROMACS and LAMMPS for MD simulations are available in the Zenodo repository (https://doi.org/10.5281/zenodo.15779813).

## Supplementary Materials

The supporting information can be downloaded at https://www.mdpi.com/article/10.3390/polym17162189/s1. The Supporting Information Section is organized as follows: Section S1 presents all details regarding the atomic partial charges of the unprotonated PDMAEMA chain as obtained from the RESP charge-fitting method. Section S2 presents the time evolution of the ${\langle R_{g}^{2}\rangle }^{0.5}$ for all systems considered in this work from MD simulations. Section S3 presents how the number of hydrogen bonds formed between the polymer and water molecules evolves with simulation time. Section S4 presents the time evolution of the SASA as well as the MD-predicted SASA values as a function of temperature for the unprotonated PDMAEMA chain. Section S5 presents how PDMAEMA chain aggregation depends on temperature for PDMAEMA solutions with *N* = 110. Section S6 presents the validation of the implementation of the quasi-harmonic entropy equation applied in this study. Section S7 presents all the *g*^inter^ (*r*) calculations carried out to study effects related to salt–polymer interactions and the degree of ionization of the PDMAEMA chain.
