# Supplementary material for: Effect of pH, Temperature, Molecular Weight and Salt Concentration on the Structure and Hydration of Short Poly(N,N-dimethylaminoethyl methacrylate) Chains in Dilute Aqueous Solutions: A Combined Experimental and Molecular Dynamics Study
Source: Polymers (Basel). 2025 Aug 10;17(16):2189. doi: 10.3390/polym17162189 (PMC12389623; doi:10.3390/polym17162189)
Supplement: Supplementary file 1 [file polymers-17-02189-s001.zip › polymers-3762043-supplementary.pdf]

Supplementary Materials

for

"Effect of pH, Temperature, Molecular Weight and Salt Concentration on the Structure and Hydration of Short Poly(N,N-dimethylaminoethyl methacrylate) Chains in Dilute Aqueous Solutions: A Combined Experimental and Molecular Dynamics Study"

Dimitris G. Mintis,[1,2] Marco Dompé,[3] Panagiotis D. Kolokathis,[2,4] Jasper van der Gucht,[3] Antreas Afantitis,[1,2,4] and Vlasis G. Mavrantzas[5,6]

[1]NovaMechanics Ltd., Nicosia 1070, Cyprus

[2] Entelos Institute, Larnaca 6059, Cyprus

[3]Physical Chemistry and Soft Matter, Wageningen University, Stippeneng 4, 6708 WE Wageningen, The Netherlands

[4]NovaMechanics MIKE, Piraeus 18545, Greece

[5]Department of Chemical Engineering, University of Patras & FORTH-ICE/HT, Patras, GR26504, Greece

[6]Particle Technology Laboratory, Department of Mechanical and Process Engineering, ETH Zürich, CH-8092 Zürich, Switzerland

S1 Details about the Coulomb parameters at zero ionization

The figure below shows the assignment of Coulomb partial charges q (e) on all atoms of an un-protonated PDMAEMA chain as obtained with the RESP charge fitting method.

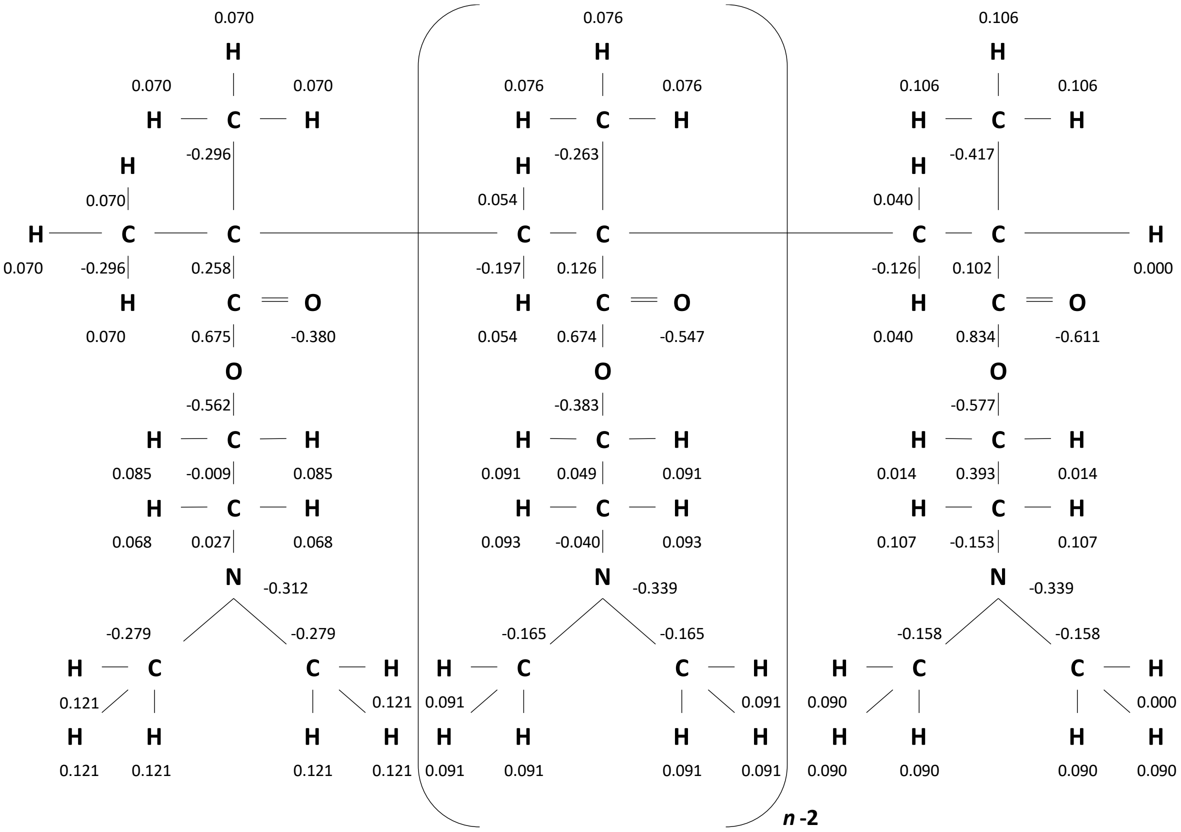

Figure S1. Partial charges computed based on the RESP charge method.

S2 Average radius of gyration versus time

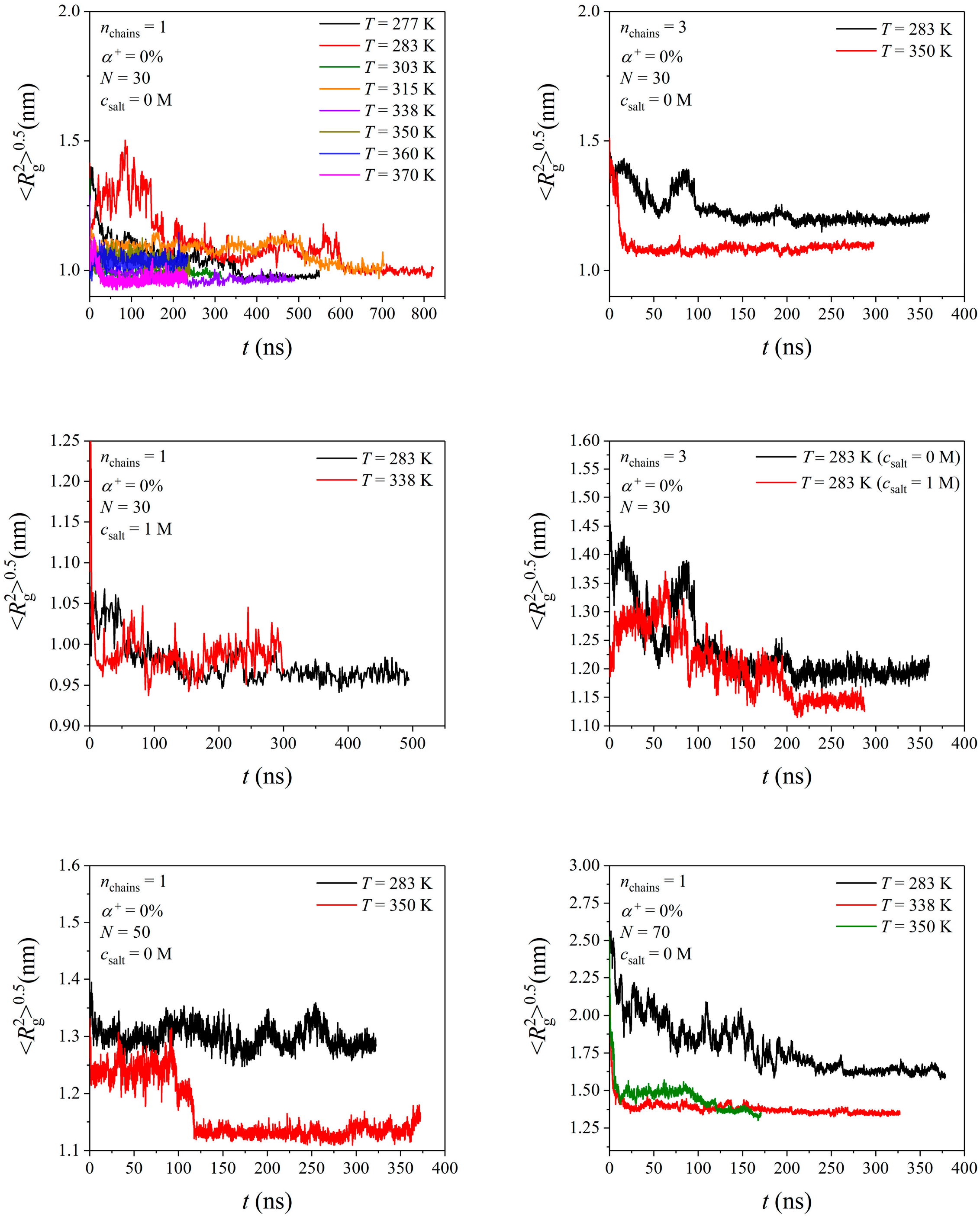

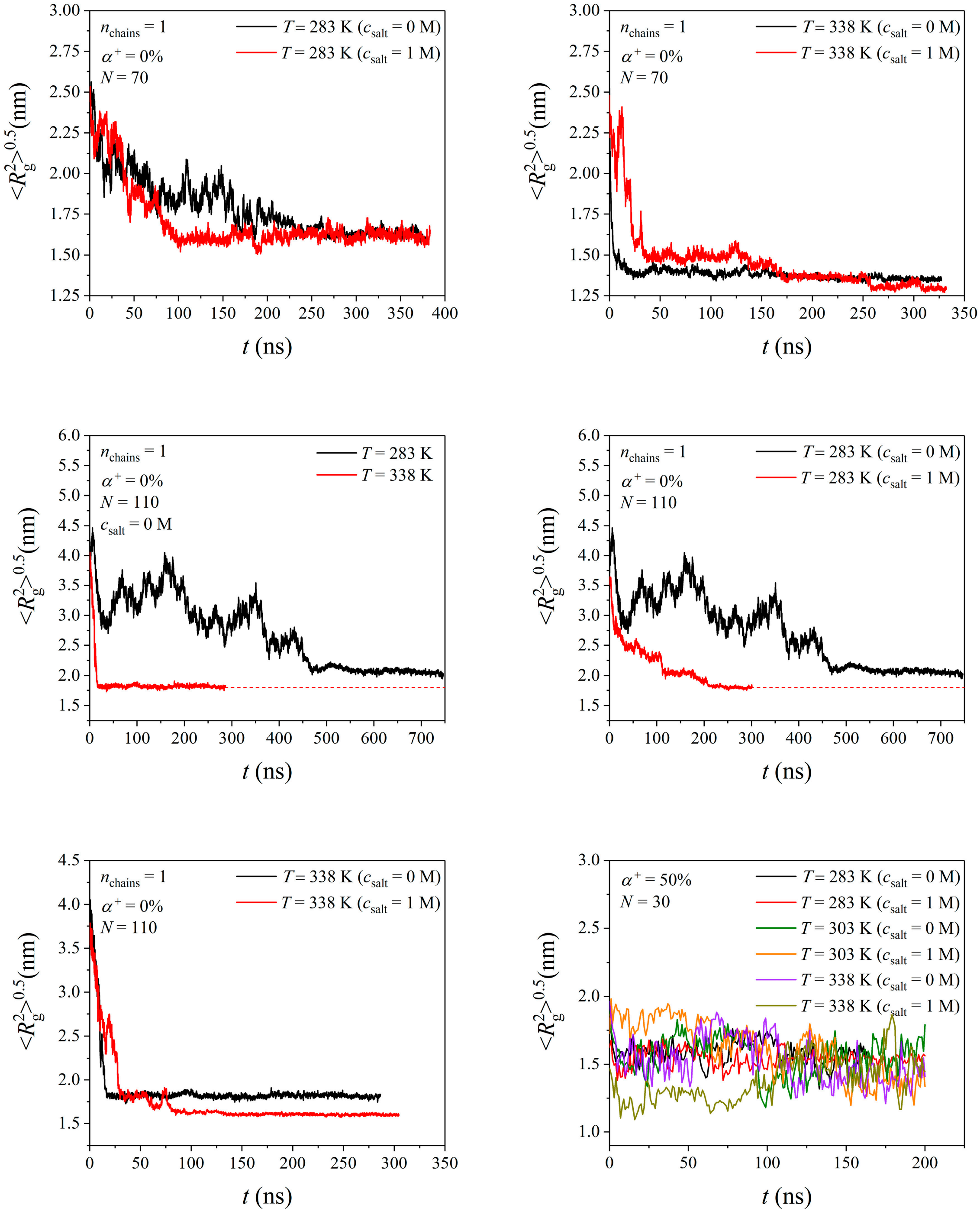
n_{chains} = 1
α^+ = 0%
N = 70
T = 283 K (c_{salt} = 0 M)
T = 283 K (c_{salt} = 1 M)
$<R_g^2>^{0.5}$(nm)
t (ns)
n_{chains} = 1
α^+ = 0%
N = 70
T = 338 K (c_{salt} = 0 M)
T = 338 K (c_{salt} = 1 M)
$<R_g^2>^{0.5}$(nm)
t (ns)
n_{chains} = 1
α^+ = 0%
N = 110
c_{salt} = 0 M
T = 283 K
T = 338 K
$<R_g^2>^{0.5}$(nm)
t (ns)
n_{chains} = 1
α^+ = 0%
N = 110
T = 283 K (c_{salt} = 0 M)
T = 283 K (c_{salt} = 1 M)
$<R_g^2>^{0.5}$(nm)
t (ns)
n_{chains} = 1
α^+ = 0%
N = 110
T = 338 K (c_{salt} = 0 M)
T = 338 K (c_{salt} = 1 M)
$<R_g^2>^{0.5}$(nm)
t (ns)
α^+ = 50%
N = 30
T = 283 K (c_{salt} = 0 M)
T = 283 K (c_{salt} = 1 M)
T = 303 K (c_{salt} = 0 M)
T = 303 K (c_{salt} = 1 M)
T = 338 K (c_{salt} = 0 M)
T = 338 K (c_{salt} = 1 M)
$<R_g^2>^{0.5}$(nm)
t (ns)

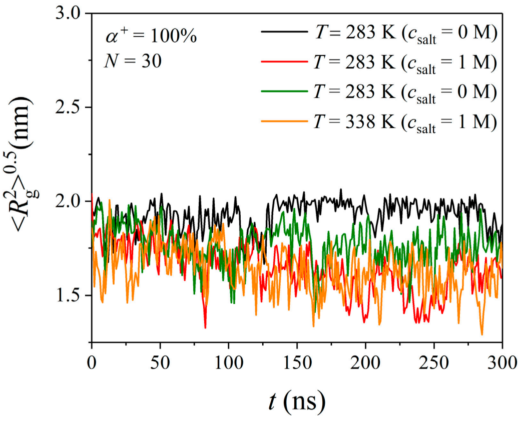

Figure S2.Time evolution of the average chain radius of gyration for all simulated systems.

S3 Number of H-bonds formed between PDMAEMA and water molecules

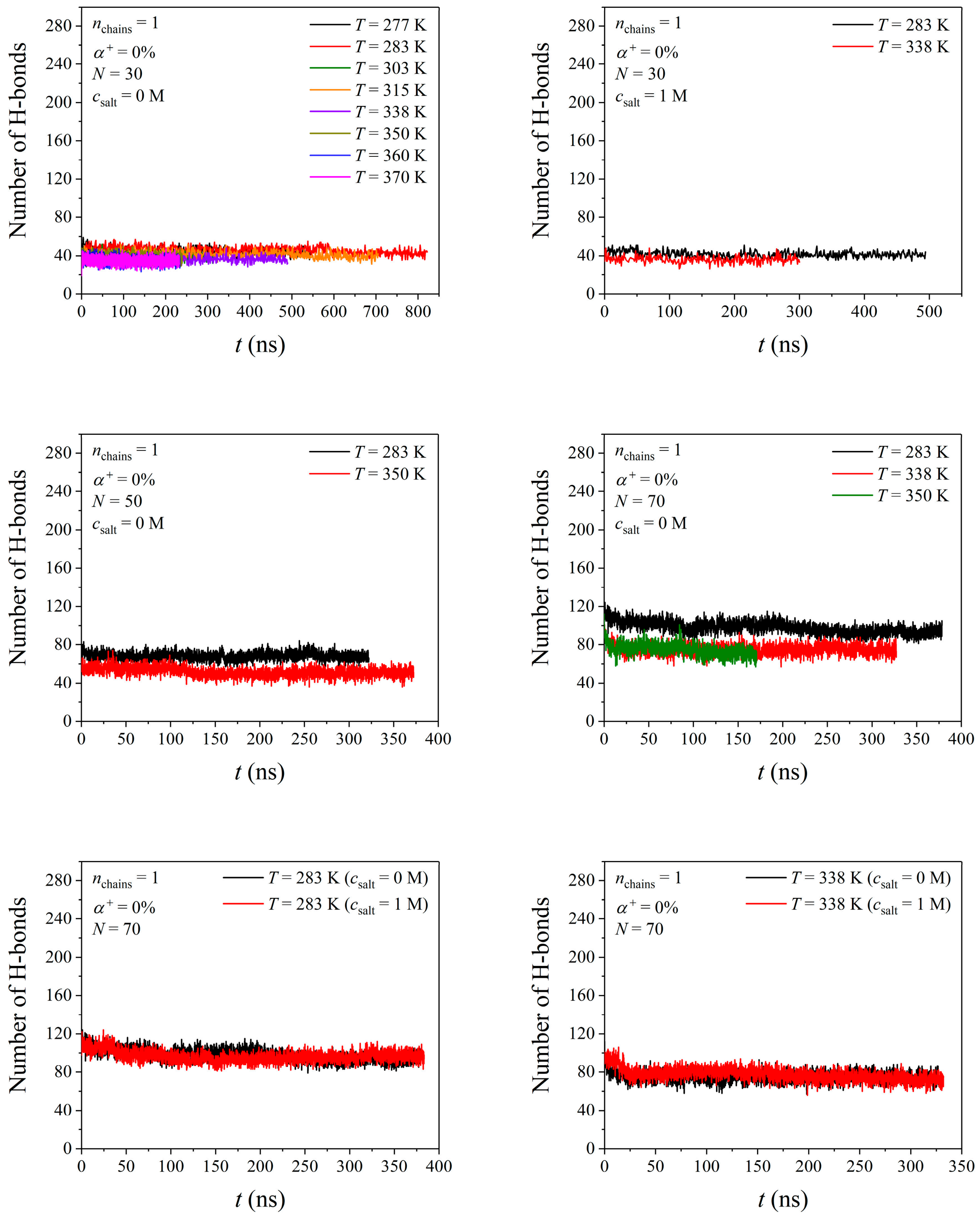

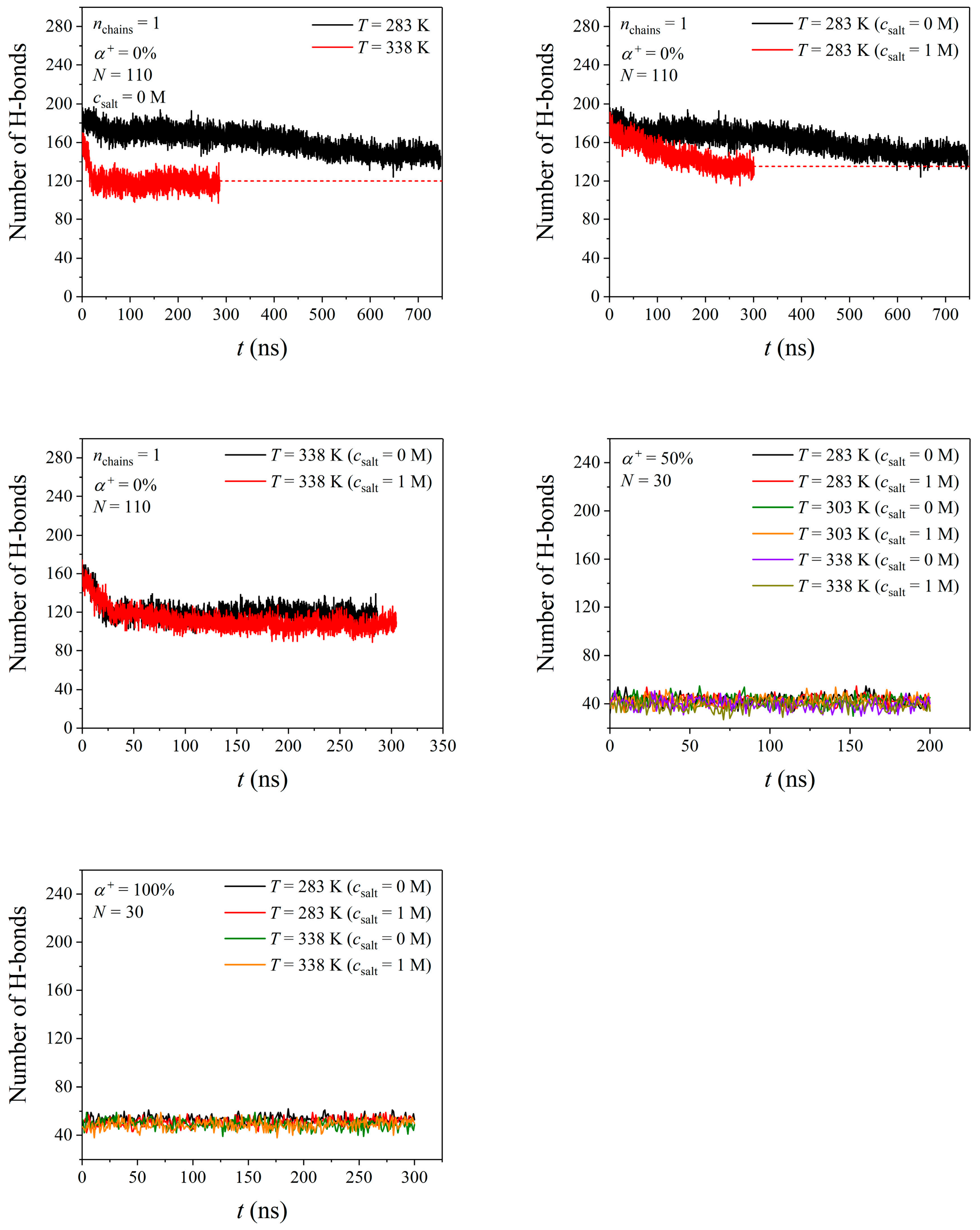

Figure S3. Time evolution of the total number of H-bonds formed between PDMAEMA and water molecules for all simulated systems.

S4 Surface accessible area (SASA)

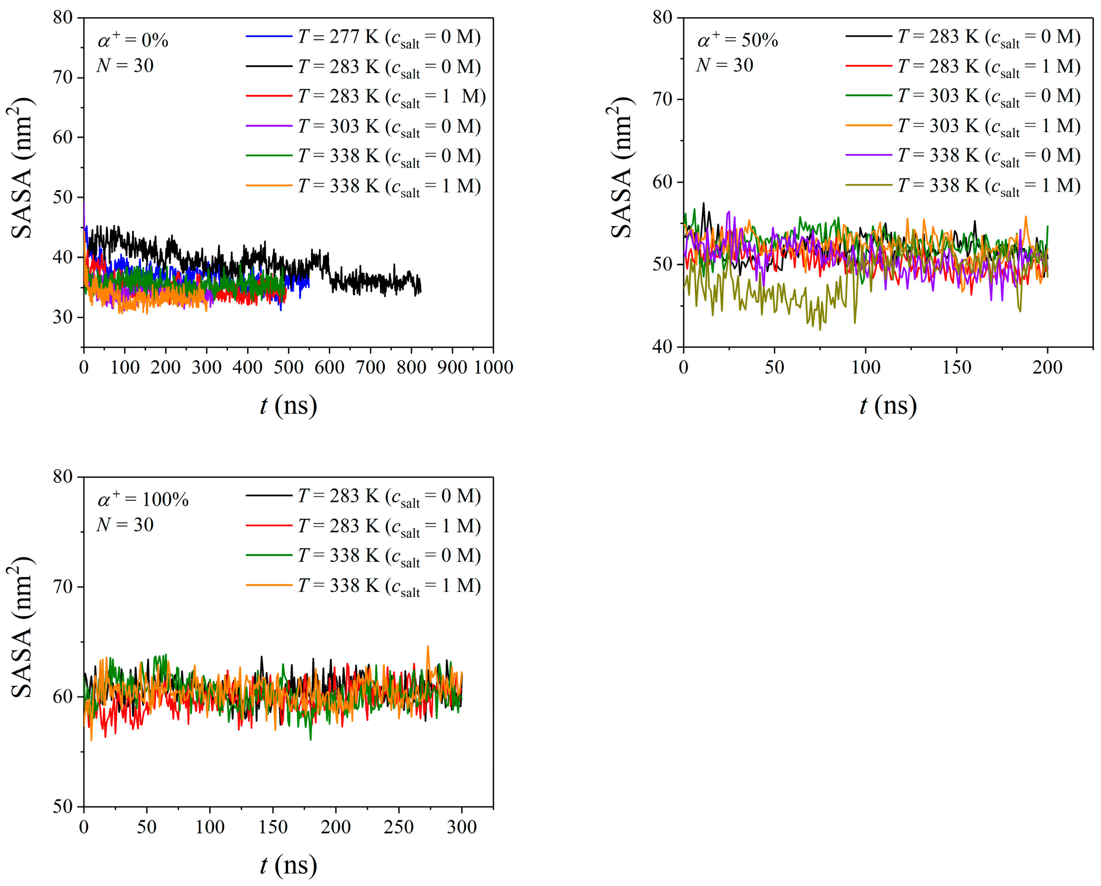

Figure S4. Time evolution of the SASA for all simulated systems.

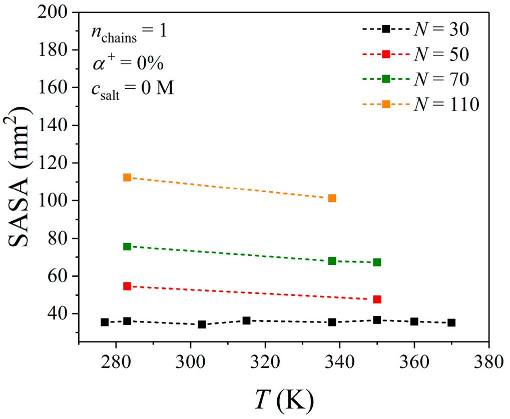

Figure S5. MD-predicted SASA values as a function of temperature for PDMAEMA solutions with different chain lengths (equivalent to $N = 30$, 50, 70, and 110).

S5 Effect of temperature on the interaction between PDMAEMA chains and their aggregation

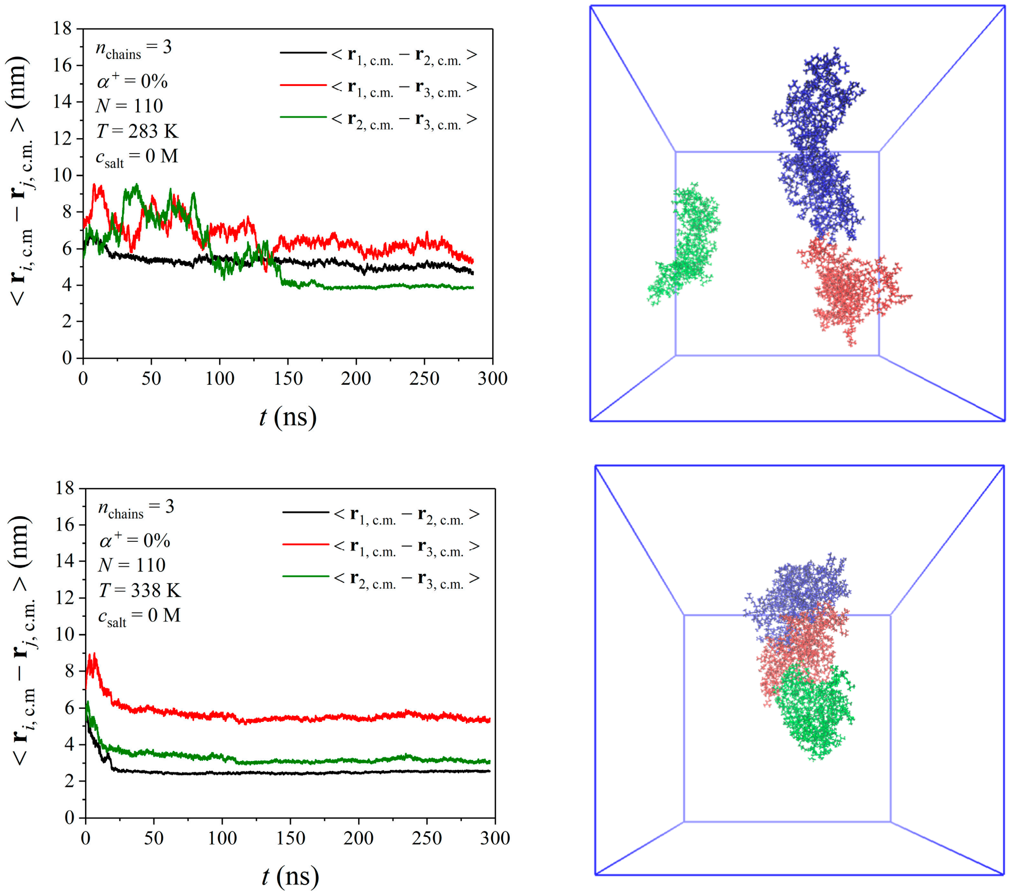

Figure S6. MD predictions for the temperature dependence of the aggregation of PDMAEMA chains in aqueous solution (chain length N = 110). The left panel shows the time evolution of the distance between the centers-of-mass of two PDMAEMA chains. The right panel shows a characteristic snapshot of the equilibrated configuration.

S6 Validation of the implementation of the quasi-harmonic entropy equation

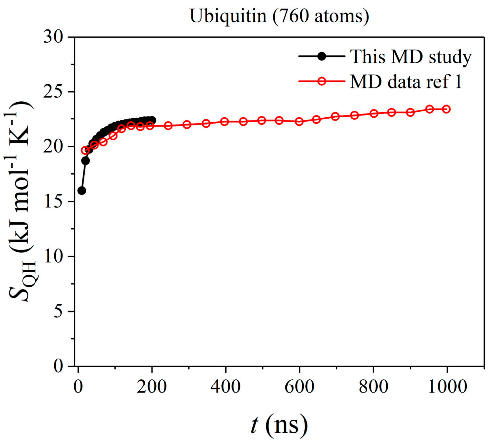

Figure S7. Validation of our implementation of the quasi-harmonic entropy equation by comparing against a previous computational study [1].

S7 Calculations of the intermolecular radial pair distribution functions

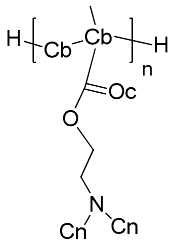

Figure S8. Notation for specific atoms located on the un-protonated PDMAEMA chain used for the calculation of the g^{inter} (r) calculations performed in this work.

S7.1 Effect of salt-polymer interactions

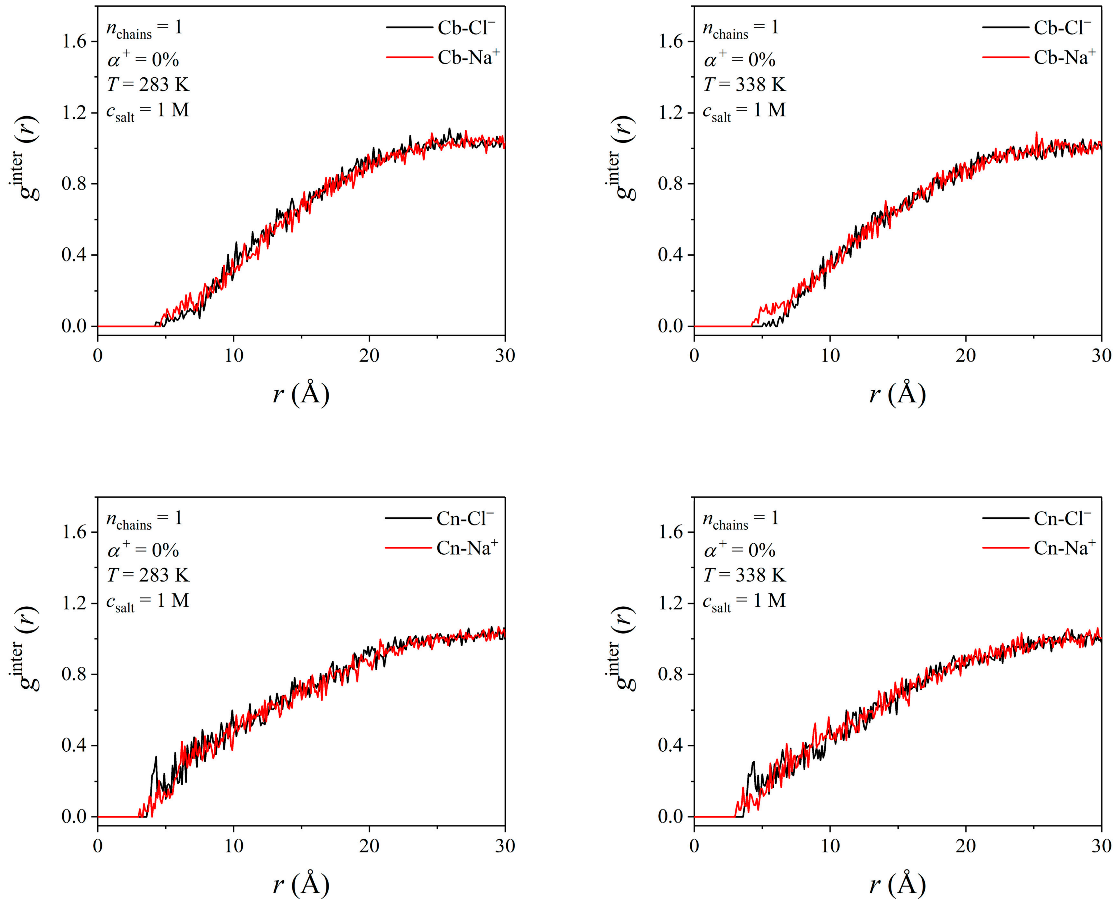

Figure S9. Radial intermolecular pair distribution function of salt anions (Cl^-) and salt cations (Na^+) with reference atoms Cb and Cn located on a PDMAEMA chain, at two different temperatures (T = 283 and 338 K).

S7.2 Effect of degree of ionization

S7.2.1 Cb-Ow

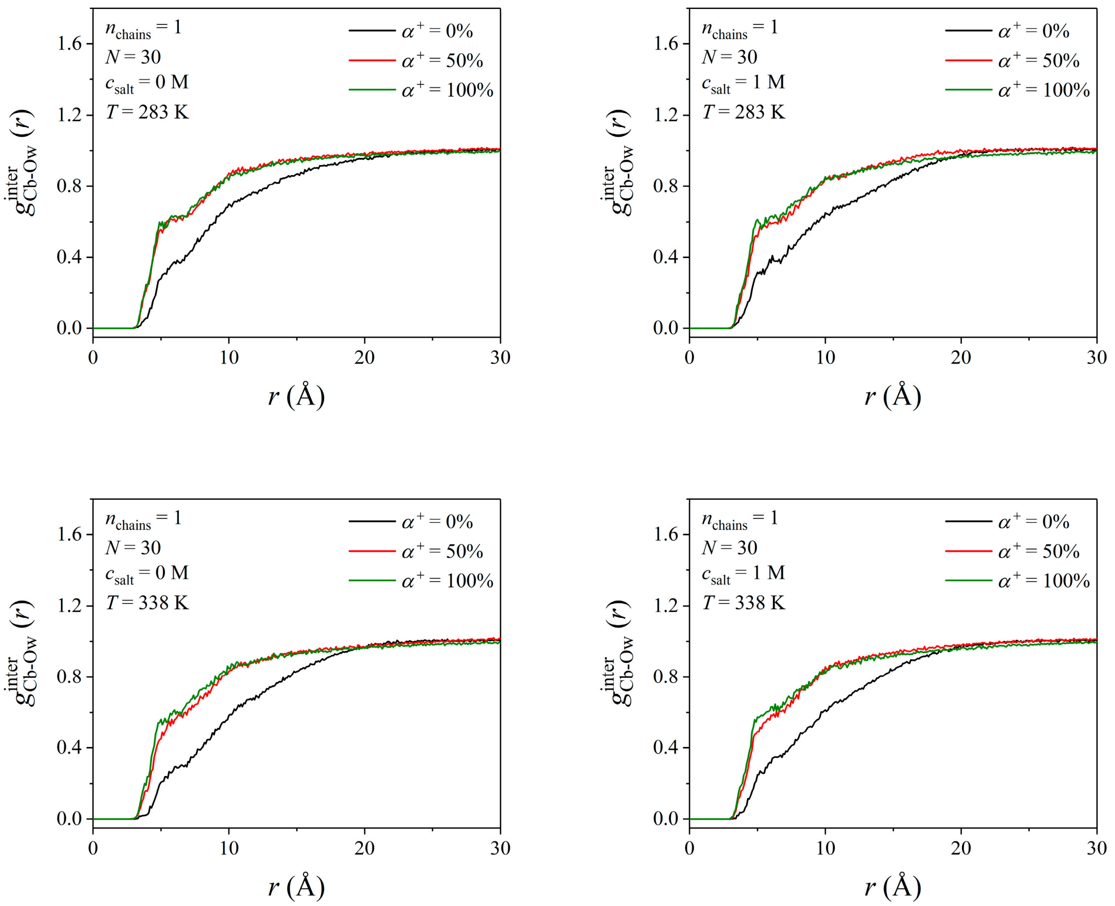

Figure S10. Effect of degree of ionization (α^+ = 0, 50, and 100 %) on the radial distribution function of Cb-Ow pairs at two different temperatures (T = 283 and 338 K) and two different salt concentrations (c_{salt} = 0 and 1 M).

S7.2.2 Oc-Ow

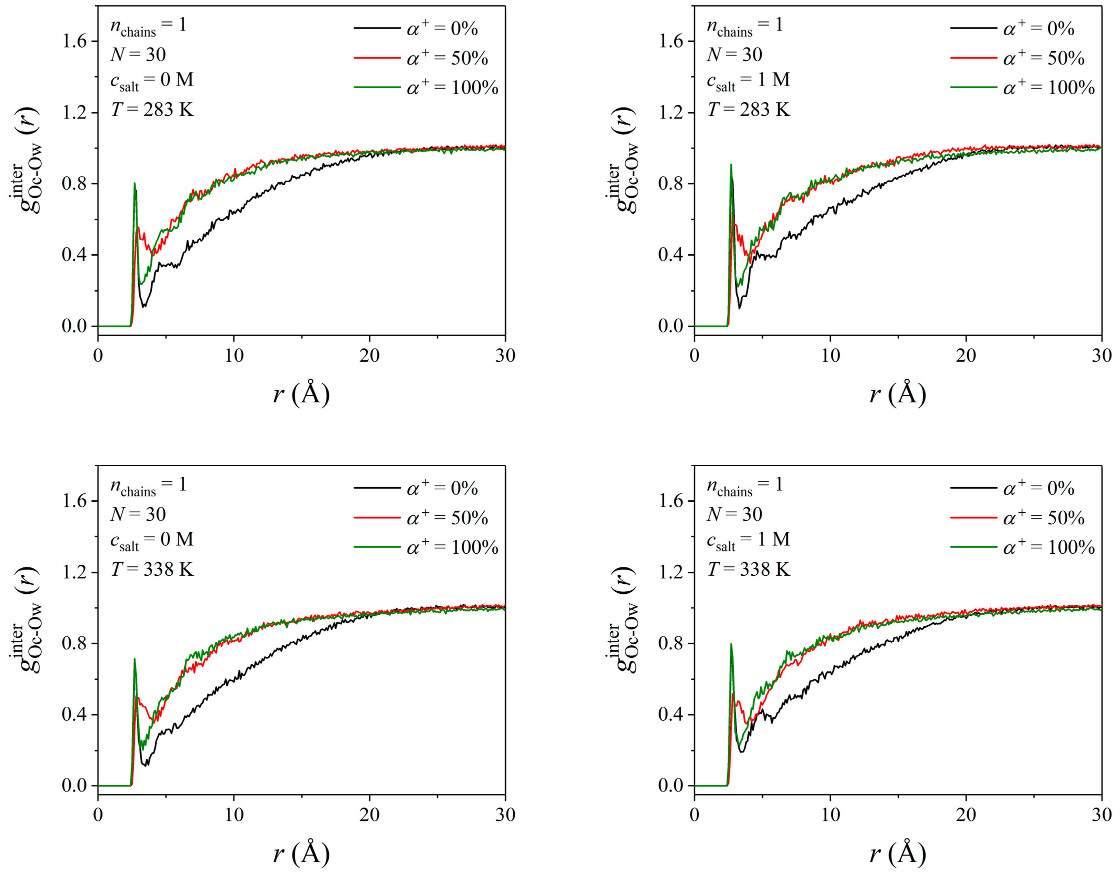

Figure S11. Effect of degree of ionization (α^+ = 0, 50, and 100 %) on the radial distribution function of Oc-Ow pairs at two different temperatures (T = 283 and 338 K) and two different salt concentrations (c_{salt} = 0 and 1 M).

S7.2.3 N-Ow

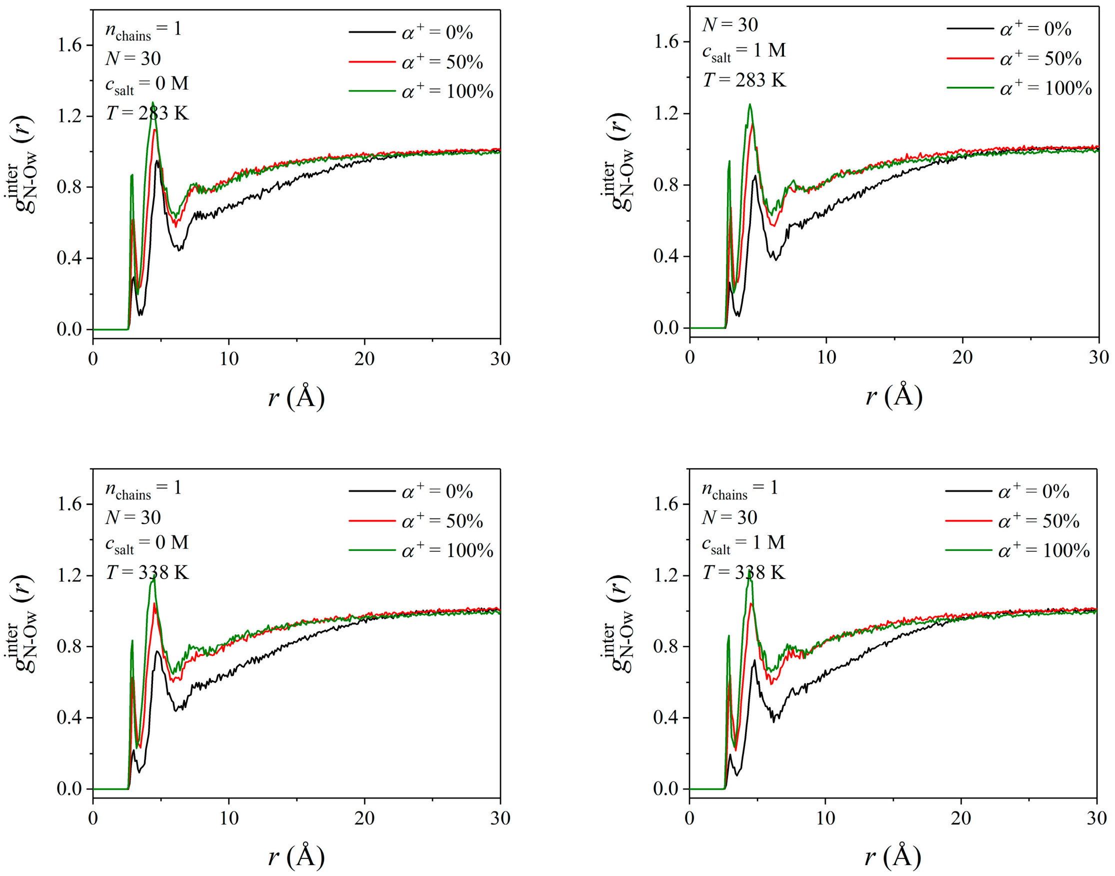

Figure S12. Effect of degree of ionization (α^+ = 0, 50, and 100 %) on the radial distribution function of pair N-Ow pairs at two different temperatures (T = 283 and 338 K) and two different salt concentrations (c_{salt} = 0 and 1 M).

Reference

1. Polyansky, A.A.; Kuzmanic, A.; Hlevnjak, M.; Zagrovic, B. On the contribution of linear correlations to quasi-harmonic conformational entropy in proteins. *Journal of Chemical Theory and Computation* **2012**, *8*, 3820-3829.
